# Exploring the Conformational Landscape and Stability of Aurora A Using Ion-Mobility Mass Spectrometry and Molecular Modeling

**DOI:** 10.1021/jasms.1c00271

**Published:** 2022-01-31

**Authors:** Lauren J. Tomlinson, Matthew Batchelor, Joscelyn Sarsby, Dominic P. Byrne, Philip J. Brownridge, Richard Bayliss, Patrick A. Eyers, Claire E. Eyers

**Affiliations:** † Centre for Proteome Research, Department of Biochemistry & Systems Biology, Institute of Systems, Molecular & Integrative Biology, University of Liverpool, Crown Street, Liverpool L69 7ZB, U.K.; ‡ Department of Biochemistry & Systems Biology, Institute of Systems, Molecular & Integrative Biology, University of Liverpool, Crown Street, Liverpool L69 7ZB, U.K.; § Astbury Centre for Structural Molecular Biology, School of Molecular and Cellular Biology, Faculty of Biological Sciences, University of Leeds, Leeds LS2 9JT, U.K.

## Abstract

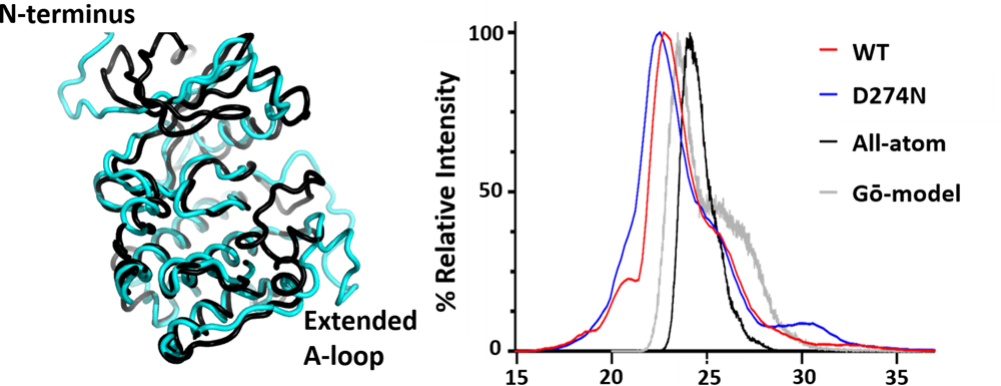

Protein kinase inhibitors are highly effective in treating diseases driven by aberrant kinase signaling and as chemical tools to help dissect the cellular roles of kinase signaling complexes. Evaluating the effects of binding of small molecule inhibitors on kinase conformational dynamics can assist in understanding both inhibition and resistance mechanisms. Using gas-phase ion-mobility mass spectrometry (IM-MS), we characterize changes in the conformational landscape and stability of the protein kinase Aurora A (Aur A) driven by binding of the physiological activator TPX2 or small molecule inhibition. Aided by molecular modeling, we establish three major conformations, the relative abundances of which were dependent on the Aur A activation status: one highly populated compact conformer similar to that observed in most crystal structures, a second highly populated conformer possessing a more open structure infrequently found in crystal structures, and an additional low-abundance conformer not currently represented in the protein databank. Notably, inhibitor binding induces more compact configurations of Aur A, as adopted by the unbound enzyme, with both IM-MS and modeling revealing inhibitor-mediated stabilization of active Aur A.

## Introduction

Protein-kinase-mediated phosphorylation permits dynamic regulation of protein function and is an essential mechanism for modulating a host of fundamental biological processes. The development of inhibitors for these enzymes has helped unravel cell signaling mechanisms and shown efficacy for the treatment of diseases such as cancer, inflammatory disorders, and diabetes,^1^ where protein phosphorylation is often dysregulated. Protein kinases consist of two lobes connected via a flexible hinge region, which forms the conserved ATP-binding site. The activation loop (A-loop) in protein kinases is 20–30 residues long with a conserved DFG motif typically extending out to an invariant APE motif. A-loops are mobile and help form a cleft that enables substrates to bind.

The majority of kinase small molecule inhibitors function by disrupting the ability of kinases to bind to and/or hydrolyze ATP and therefore block phosphate transfer to protein substrates, either by competing directly with ATP binding or by locking the enzyme in an “inactive” conformation. Understanding the selectivity and specificity of these small molecule inhibitors toward target enzymes is critical for correct interpretation of data arising from their use. “Off-target” effects driven through conserved binding sites within similar kinase conformations can exist across members of the evolutionary-related kinome.^2^

Protein kinase inhibitors are broadly classified based on their ability to bind to different regions within the enzyme superfamily or to a specific conformational state. While “type I” inhibitors, such as staurosporine and dasatanib, competitively bind to the ATP-binding site of kinases in the active “DFG-in” conformation, “type II” inhibitors like imatinib are “mixed mode”, contacting both the ATP-binding site and an adjacent hydrophobic pocket that is only accessible in the “DFG-out” conformation, which serves to lock the target kinase into an inactive state.^3−5^ In addition to significant diversity in the DFG-in and (in particular) DFG-out structures of multiple kinases, some can adopt an “intermediary” orientation between the typical DFG-in and DFG-out conformation, termed DFG-up or DFG-inter.^6^

Characterizing the effects of small molecule inhibitors on the structure and catalytic activity of protein kinase targets and the influence of post-translational modifications (PTMs; typically activating phosphorylation) on these interactions provides fundamental mechanistic knowledge for drug discovery and helps iterative drug design. However, limitations often arise with crystallographic structural studies, with some protein–inhibitor complexes being intransigent to crystallization.^7^ Moreover, conformational dynamics and the effects of cofactor and/or small molecule binding are constrained in protein crystals. While NMR can be used to examine conformational flexibility, obtaining a full atomic map for proteins or complexes greater than ∼50 kDa remains a challenge.^8^ Native mass spectrometry (MS), in which liquid-phase samples are subjected to electrospray ionization (ESI) under nondenaturing conditions that closely mimics their physiological environment, is increasingly being used to investigate the topology of intact protein complexes. Under carefully controlled conditions (pH, ionic strength, applied voltage, gas pressure), the native state of the analyte protein (and ligand) complexes can be maintained.^9^ Native MS is primarily used to define the molecular mass of protein complexes (and component stoichiometry) as well as compare the relative dissociation contant (*K*_D_) of ligand binding and protein complexes^10−15^ and, in a broad sense, the degree of protein “disorder”.^16^ When used in combination with ion mobility (IM) spectrometry, native MS (IM-MS) can reveal structural changes that arise due to ligand binding or protein modification as well as interrogate protein conformational dynamics, stability, and unfolding transitions.^17,18^ When appropriately calibrated, native IM-MS can also be used to determine the rotationally averaged collision cross section (CCS) of proteins and their complexes, empirical information that can be compared both with other structural measurements and theoretical calculations^12,19^ to understand the effects of PTMs or small molecule binding on protein structure and dynamics.

The Ser/Thr protein kinase Aurora A (Aur A) is associated with mitotic entry and plays critical roles in centrosome maturation and separation.^20^ In the early G-phase of the cell cycle, Aur A is recruited to the centrosomes where it facilitates spindle assembly.^21,22^ Later, Aur A microtubule association and activation requires binding of the Eg5-associated microtubule factor TPX2.^23−25^ The N-terminus of TPX2 binds to the Aur A catalytic domain, inducing conformational changes in the kinase, which both enhances autophosphorylation at Thr288 within the activation loop and shields this activating phosphorylation site from phosphatases.^26−28^ Overexpression of Aur A results in mitotic abnormalities and the development of tetraploid cells.^29^ While elevated levels of Aur A are broadly associated with a range of cancers, including breast, colorectal, ovarian, and pancreatic,^30^ abolition of Aur A activity leads to abnormalities in mitotic spindle assembly, resulting in spindle checkpoint-dependent mitotic arrest.^31−34^ More recently, catalytically independent roles for Aur A in different phases of the cell cycle have also been described.^33,35−38^

Many Aur A inhibitors have been reported over the last two decades,^39^ a number of which target all three members of the Aurora kinase family, such as VX-680/tozasertib.^34,40^ Inhibitors that show a preference between human Aurora kinases have also been developed by targeting specific amino acid differences in the ATP site that occur between Aur A and Aur B/C.^41−43^ For example, alisertib (MLN8237) is a selective Aur A inhibitor that, alongside the earlier tool compound MLN8054,^44,45^ has been characterized using a variety of *in vitro* and *in vivo* preclinical models.^46^ Importantly, MLN8237 has been phenotypically target-validated in cells with drug-resistant Aur A alleles^37,45^ and has demonstrated efficacy in a number of human cell lines and tumor models.^47^ MLN8237 has been assessed in Phase I and II clinical trials for hematological malignancies, patients with advanced solid tumors, and children with refractory/recurrent solid tumors.^48,49^

To better understand the effects of small molecule binding on Aur A, Levinson and colleagues recently evaluated the conformational effects of a panel of clinically relevant Aur kinase inhibitors across different activation states of Aur A using time-resolved Förster resonance energy transfer (TR-FRET). Using this approach, they were able to track dynamic structural movements of the A-loop, distinguishing between inhibitors that induce DFG-in states from compounds that promote other conformations (DFG-out/DFG-up/DFG-inter). The TR-FRET data was consistent with equilibrium shifts toward three distinct conformational groups, including DFG-in, DFG-out, and “unique” structural states.^50^

In this study, we employ IM-MS to explore the effects of inhibitor binding on the conformational landscape, dynamics, and stability of two variants of the Aur A kinase domain: a catalytically active phosphorylated protein and an inactive nonphosphorylated version created by a point mutation within the DFG motif (D274N). These studies reveal differences in the conformational landscape adopted by Aur A upon activation and in the presence of inhibitors, with active Aur A being less conformationally flexible. Furthermore, our data also suggest that chemical inhibitors induce stabilization of Aur A, revealing differences in intermediate partially unfolded conformations that correlate with previously reported DFG-in/out/up classifications with distinct compounds. Together, our biophysical data demonstrate the applicability of IM-MS for distinguishing modes of inhibitor binding to kinases that could be extendable to other members of this highly druggable superfamily.

## Materials and Methods

### Protein Purification

6His-N-terminally tagged human Aur A (122–403) wild-type (WT) or D274N was individually expressed from a pET30-TEV vector in BL21 (DE3) pLysS *Escherichia coli* (Novagen), with protein expression being induced with 0.4 mM IPTG for 18 h at 18 °C. *E. coli* pellets were lysed in 100 mL of ice cold lysis buffer (50 mM Tris-HCl pH 7.4, 10% glycerol, 300 mM NaCl, 10 mM imidazole, 1 mM DTT, 100 mM EDTA, 100 mM EGTA, protease inhibitor tablet (Roche)). The lysed cells were then sonicated on ice using a 3 mm microprobe attached to an MSE Soniprep 150 Plus motor unit at an amplitude of 16 μm in 30 s intervals. Samples were centrifuged for 1 h at 8 °C (43 000*g*) to pellet the cellular debris and then filtered through a 0.22 μm filter. His-tagged Aur A was separated from clarified bacterial cell lysate using a Nickel HisTrap HP column and pre-equilibrated in wash buffer (50 mM Tris-HCI pH 7.0, 10% glycerol, 300 mM NaCl, 20 mM imidazole, 1 mM MgCl_2_). After the cell lysate was loaded, the column was washed with 10 mL of wash buffer, followed by 10 mL of elution buffer (50 mM Tris-HCI pH 7.0, 10% glycerol, 300 mM NaCl, 500 mM imidazole, 1 mM MgCl_2_, 1 mM DTT), and the His-tag was cleaved by addition of 25 μg of TEV protease and incubation for 18 h at 4 °C. Subsequently, Aur A was further purified using a Superdex 200 16 600 column (GE Healthcare) attached to an AKTA FPLC system and a Frac-920 (GE Healthcare), which was equilibrated in filtered and degassed gel filtration buffer (20 mM Tris pH 7.0, 10% glycerol, 200 mM NaCl, 40 mM imidazole, 5 mM MgCl_2_, 1 mM DTT). Aur-A-containing fractions were pooled and passed through a HisTrap column to remove residual non-TEV cleaved material. Samples were stored in small aliquots at −80 °C prior to further analysis.

### Native Ion-Mobility Mass Spectrometry

Immediately prior to native MS analysis, purified Aur A proteins were buffer-exchanged into 150 mM NH_4_OAc using an Amicon spin filter (10 kDa cutoff). Spin columns were prewashed with 500 μL of 150 mM NH_4_OAc prior to the addition of protein and spun 3× for 10 min at 13 000 rpm. Following the final spin, the filter was inverted into a new collection tube and spun for 2 min at 3000 rpm to collect the protein. Protein concentration was calculated using a NanoDrop spectrophotometer (280 nm) and adjusted to 5 μM for MS analysis. To evaluate the effect of small molecule binding, Aur A proteins were incubated with 4% DMSO (vehicle control) or a 10× molar excess of inhibitor or TPX2-activating 43-mer peptide (H_2_N-MSQVKSSYSYDAPSDFINFSSLDDEGDTQNIDSWFEEKANLEN-CONH_2_, Pepceuticals) and equilibrated for 10 min at room temperature prior to IM-MS analysis. Ion-mobility mass spectrometry data was acquired on a Waters Synapt G2-Si instrument operated in “resolution” mode. Proteins were subject to nanoelectrospray ionization (nESI) in positive ion mode (at ∼2 kV) with a pulled nanospray tip (World Precision Instruments 1B100-3) prepared as detailed in ref (51). Ions of interest were mass-selected in the quadrupole prior to IMS. The pressure in the TWIMS cell was set at 2.78 mbar (nitrogen), with an IM wave height of 23 V, a wave velocity of 496 m/s, and a trap bias of 33.

### Collision-Induced Unfolding

For collision-induced unfolding (CIU) experiments, the 11+ charge state of Aur A (WT or D274N) in the absence or presence of bound inhibitor was quadrupole-isolated and subjected to collisional activation by applying a CID activation in the ion trap of the TriWave. The activation voltage was increased gradually from 16 to 34 V in 2 V intervals before IMS measurement. CIU was carried out with a traveling-wave height of 27 V, velocity of 497 m/s, and trap bias of 35.

### Phosphosite Mapping

Purified Aur A was buffer-exchanged into 100 mM ammonium bicarbonate, reduced with 4 mM DTT (30 min, 60 °C) and reduced Cys residues alkylated with 7 mM iodoacetamide (45 min, dark at room temperature), as described previously.^52^ Proteins were then digested with trypsin (2% (w/w) Promega) for 18 h at 37 °C. RapiGest SF hydrolysis was carried out using 1% TFA (1 h, 37 °C, 400 rpm), prior to LC/MS/MS analysis.^52^ Raw mass spectrometry data files were processed with Proteome Discoverer (v2.4). Data was searched using MASCOT (2.6) against a human UniProt Aur A database limited to residues 122–403 or the D274N mutation. Parameters were set as follows: MS1 tolerance of 10 ppm, MS2 mass tolerance of 0.01 Da; enzyme specificity was defined as trypsin with two missed cleavages allowed; carbamidomethyl Cys was set as a fixed modification; Met oxidation and Ser/Thr/Tyr phosphorylation were defined as variable modifications.

Data were filtered to a 1% false discovery rate (FDR) on peptide spectrum matches (PSMs) using automatic decoy searching with MASCOT. ptmRS node with Proteome Discoverer was used to determine phosphosite localization confidence.

### CCS Calibration and IM-MS Data Analysis

Calibration of the TriWave device was performed using β-lactoglobulin (Sigma L3908), cytochrome c (Sigma C2506), and bovine serum albumin (Sigma A2153) as previously described.^53^ All data were processed using MassLynx (v. 4.1) and Origin (Version 2021b) to determine collision cross section (CCS) values. Average IM-MS profiles from three replicate analyses were used for Gaussian fitting of Aur A individual conformational states using Origin (Version 2021b). The Fit Peaks Pro function was implemented to initially assign the most abundant peak (conformer II). Additional peaks were added for conformers I, III, and IV, where applicable. The CCS, CCSD, and area parameters for each assigned conformer were manually adjusted using Fit Control and the iteration feature, in order to obtain the best fit between experimental data (black line) and sum of Gaussians (red line). Black error bars are representative of the SD, and the error between the experimental line and sum of Gaussians was reported with *R*^2^. Scatter plots of ^TW^CCS_N2>He_ (nm^2^) values versus CCS distribution (CCSD) (nm^2^) were generated using ggplot in RStudio. CIU unfolding plots were generated using CIUSuite 2.^54^

### Western Blotting

Samples were heated to 95 °C for 5 min in sample buffer (50 mM Tris pH 6.8, 1% SDS, 10% glycerol, 0.01% Bromophenol Blue, 10 mM DTT) prior to separation on a 10% polyacrylamide gel and then transferred onto nitrocellulose membrane. Western blotting was carried out using standard procedures. Nitrocellulose membranes were blocked in 5% milk powder (Marvel) in Tris-buffered saline and 0.1% Tween 20 (TBST) (20 mM Tris pH 7.6, 137 mM NaCl, 0.1% Tween-20 (v/v)) for 1 h at room temperature on a shaking rocker. All antibodies were prepared in 5% milk TBST. Antiphospho Aur A (T288) (Cell Signaling Technologies 2914) was used at a 1:5000 dilution and incubated with the membrane for 18 h at 4 °C, as described previously.^55^ Secondary antirabbit antibody (1:5000) was incubated for 1 h at room temperature. X-ray film was exposed to the membrane following application of Immobilon Western Chemiluminescent HRP Substrate (Millipore) developing reagent. The films were developed using an ECOMAX X-ray film processor (Protec).

### Protein Kinase Activity Assays

*In vitro* peptide-based Aur A assays were carried out using a Caliper LapChip EZ Reader platform (PerkinElmer), which monitors real-time phosphorylation-induced changes in the mobility of a fluorescently labeled Kemptide peptide substrate (5′-FAM-LRRASLG-CO_NH2_).^56^ The activity of both WT and D274N Aur A variants (10 ng) was evaluated by incubation with 1 mM ATP and phosphorylation of 2 μM fluorescent peptide substrate in 50 mM HEPES (pH 7.4), 0.015% (v/v) Brij-35, 1 mM DTT, and 5 mM MgCl_2_. The activity of Aur A after incubation with TPX2 peptide (5 μM) was determined using a TPX2 concentration range of 0.0004–40 μM. To confirm the loss of catalytic activity, D274N Aur A was also assayed with 40 μM TPX2 peptide. Data was plotted as % peptide conversion (phosphorylation) over a linear real-time scale, using GraphPad Prism software as described in ref (57).

### Differential Scanning Fluorimetry (DSF) Assays

Thermal shift assays were performed using a StepOnePlus Real-Time PCR machine (Life Technologies) with Sypro-Orange dye (Invitrogen) and thermal ramping (0.3 °C per min between 25 and 94 °C). All proteins were diluted to 5 μM in 50 mM Tris–HCl (pH 7.4) and 100 mM NaCl in the presence of 40 μM inhibitor [or 4% (v/v) DMSO as the vehicle control concentration], 1 mM ATP, and/or 10 mM MgCl_2_. Data was processed using the Boltzmann equation to generate sigmoidal denaturation curves, and average *T*_m_/Δ*T*_m_ values were calculated using GraphPad Prism software, as previously described.^58^

### Molecular Modeling

Missing parts of the Aur A sequence were modeled into crystal structures using the PyMod plugin^59^ in PyMOL.^60^ Homology models of the full 122–403 catalytic domain sequence (equivalent to the catalytic domain) were built using MODELER^61^ based on the following Aur A crystal structures: 1MUO, 1OL5, 1OL6, 1OL7, 2WTV (chain A and B), 3E5A, 4C3P, 4CEG, 4J8M, 4JBQ, 5EW9, 5G1X, 5L8K, 5ODT, 6HJK (Table S1). Where present in the crystal structure, phosphorylated residues and the D274N substitution were accounted for, but otherwise, the amino acid sequence was the same as UniProt human Aur A accession O14965. All other bound proteins or ligands were removed. The MODELER loop modeling function in PyMod was then used to build 10 improved Aur A models, allowing only the newly added residues of the N- and C-termini (and any new A-loop residues) to change. The model with the lowest “objective function” and without obvious new contacts made with the rest of the protein was chosen as the starting structure for modeling.^33^ All-atom simulations were performed with the CHARMM36m force field^62^ using NAMD.^63^ Inputs for NAMD simulations were generated using CHARMM-GUI^64^ based on the PYMOD-generated models. Phosphorylated threonine residues use the doubly deprotonated pThr patch (THPB). N- and C-termini were uncapped. The protein was solvated in a rectangular waterbox with a minimum distance of 10 Å between the protein and the box edge (∼20 000 TIP3P water molecules). Cl^–^ ions were added to neutralize the protein. Solvated structures were first subjected to 10 000 conjugate gradient energy-minimization steps. Prior to the collection of trajectory data, a heating protocol that raised the temperature of the system from 0 to 300 K over 60 000 steps and a short pre-equilibration at 300 K for 125 000 steps were used. The time step of 2 fs was used throughout. Trajectory frames were recorded every 5000 steps (10 ps), and simulations ran for >300 ns with temperature controlled at 300 K and pressure at 1 atm using Langevin dynamics.

Simulation trajectories were processed and analyzed using Wordom.^65^ The protein component of the system was isolated and aligned, and individual trajectory frames were extracted for CCS measurements. IMPACT^66^ was used to estimate CCS values for protein crystal structures and trajectory snapshots. The default atomic radii and convergence parameters were used for all-atom simulations. In all cases, the raw IMPACT CCS value based on projection approximation (rather than the recalibrated TJM value) was used, as this provided much better comparison with experimental data for Aur A models and for a bovine serum albumen test model. DFG-motif conformations were described using criteria introduced by Modi and Dunbrack.^6^ Distances were measured from DFG-Phe to a conserved residue position in the C-helix (D1 = F275-Cζ to Q185-Cα, for Aur A) and from DFG-Phe to the important salt-bridge forming Lys on the β3 strand (D2 = F275-Cζ to K162-Cα). The correlation between D1 and D2 indicates whether the structure exhibits a DFG-in, DFG-out, or DFG-up/inter conformation.

Go̅-like models and potentials were generated from all-atom initial structures using the MMTSB web service (https://mmtsb.org/webservices/gomodel.html).^67,68^ MD simulations of Go̅-like models were carried out using Langevin dynamics and the CHARMM package, version 44/45.^69^ The time step was 10 or 15 fs. Simulations across a range of different temperatures were performed to gauge where the unfolding transition occurs, and then, production simulations were performed below this temperature. For Go̅-like models, the atomic radius used to generate CCS values in IMPACT was the average distance between each Cα atom (3.8 Å). This provided reasonable comparison with the all-atom simulation results.

Clustering of Go̅-like model conformers was performed using a 15 Å RMSD cutoff value between clusters. To gauge local flexibility, root-mean-square fluctuation (RMSF) values for each residue in Go̅-like model simulations were calculated with respect to an ensemble-averaged structure. An analysis of native contacts in Go̅-like models was performed as described by Karanicolas and Brooks,^70^ using a low-temperature (250 K) simulation to define native contact distances at 80% occupancy.

## Results and Discussion

### WT but Not D274N Aur A Is Phosphorylated and Catalytically Active

To evaluate the effects of phosphorylation and small molecule binding on the conformational landscape, dynamics, and flexibility of Aur A, we expressed and purified two well-studied Aur A catalytic domain (amino acids 122–403) variants from *E. coli*: a wild-type (WT) active version that extensively autophosphorylates during exogenous expression (and exhibits reduced electrophoretic mobility dependent on phosphorylation during SDS-PAGE), and a noncatalytically active variant (D274N), in which the essential DFG-motif Asp is replaced with Asn (Figure S1A).^23^ MS/MS-based phosphorylation site mapping of this purified WT Aur A (122–403) revealed at least six sites of autophosphorylation, including Thr288, which lies in the kinase activation loop and is the classical biomarker for Aur A catalytic activity (Figure S1B,C).^26,52,71,72^ No autophosphorylation sites were observed in D274N Aur A, and this was confirmed by immunoblotting with a phosphospecific antibody against pThr288 (Figure S1B). Enzymatic assays confirmed that WT, but not the D274N variant of Aur A, exhibited robust catalytic activity toward the substrate peptide in the presence of ATP and Mg^2+^ ions (Figure S1D), as expected.

**Figure 1:**
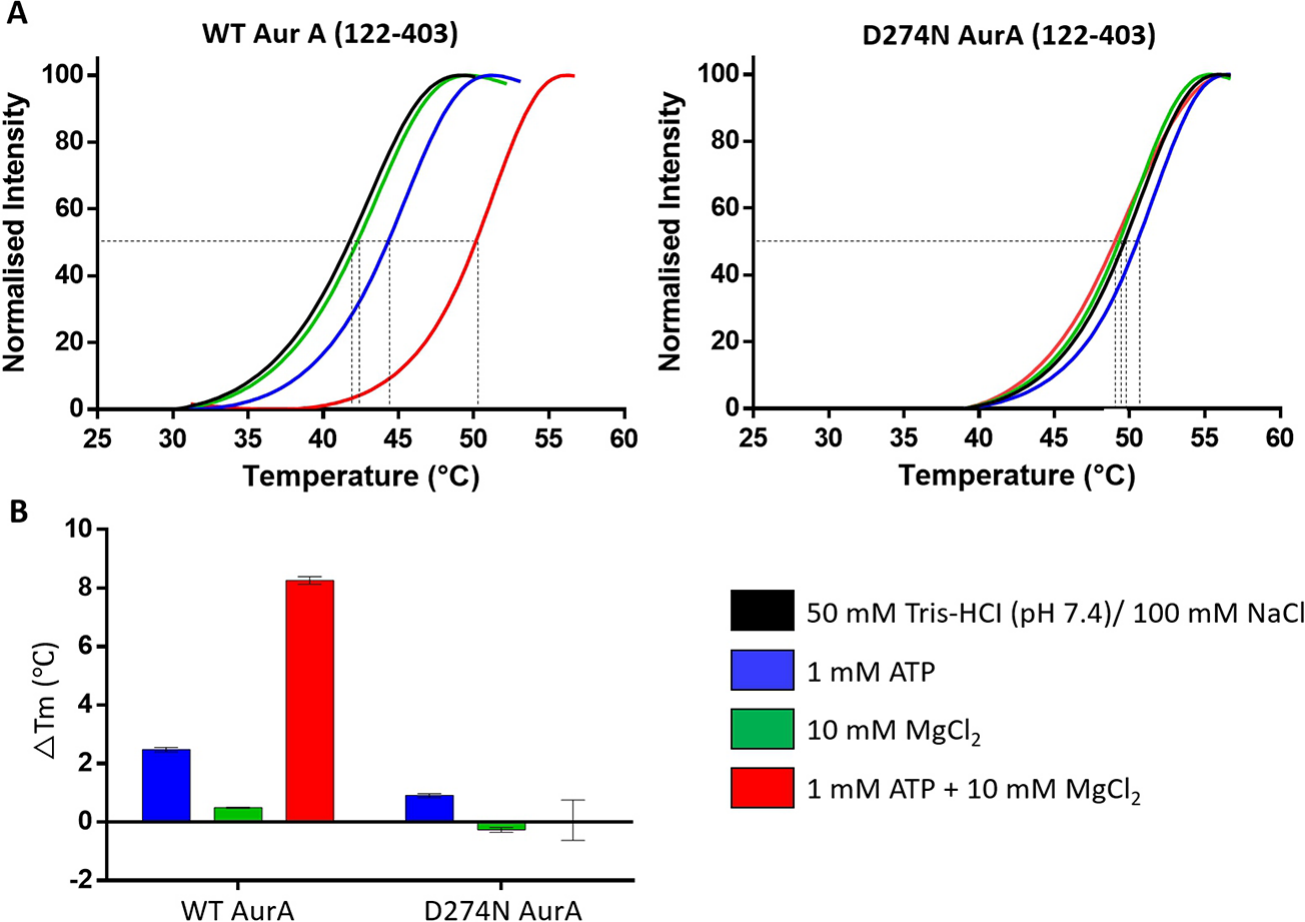
Wild-type (WT) phosphorylated Aur A (122–403) is less thermodynamically stable than a catalytically inactive nonphosphorylated D274N Aur A (122–403) variant. (A) DSF thermal stability assay with 5 μM Aur A (black), in the presence of 1 mM ATP (blue), 10 mM MgCl_2_ (green), or 1 mM ATP + 10 mM MgCl_2_ (red). (B) Difference in melting temperature (Δ*T*_m_) compared with buffer control is presented for both WT and D274N Aur A (122–403).

The thermal unfolding profile of WT Aur A, reported as the *T*_m_ value measured by differential scanning fluorimetry (DSF), increased markedly in the presence of Mg^2+^/ATP (+8.3 °C), indicative of tight ATP binding, as previously reported.^73^ In contrast, there was negligible change (+0.1 °C) in the calculated *T*_m_ of D274N Aur A under the same conditions (Figure 1B), consistent with the inability of this protein to bind Mg^2+^/ATP. Stabilization was greatly reduced for the WT protein in the presence of ATP alone (Δ*T*_m_ = +2.5 °C), which is supportive of previous studies showing that Mg^2+^ is required for high-affinity binding of ATP to Aur A.^73^ The lower melting temperature of WT Aur A compared with D274N Aur A also suggests that inactive D274N Aur A is more stable than the active phosphorylated form (Figure 1A).

### Active Phosphorylated Aur A Is Less Stable and Less Conformationally Dynamic than the Inactive Enzyme

To assess the effects of phosphorylation on the structure and conformational flexibility of Aur A, we analyzed phosphorylated WT and nonphosphorylated D274N proteins by native IM-MS, using traveling-wave ion mobility spectrometry (TWIMS) to determine the rotationally averaged collision cross section (^TW^CCS_N2→He_) following drift time calibration.

**Figure 2:**
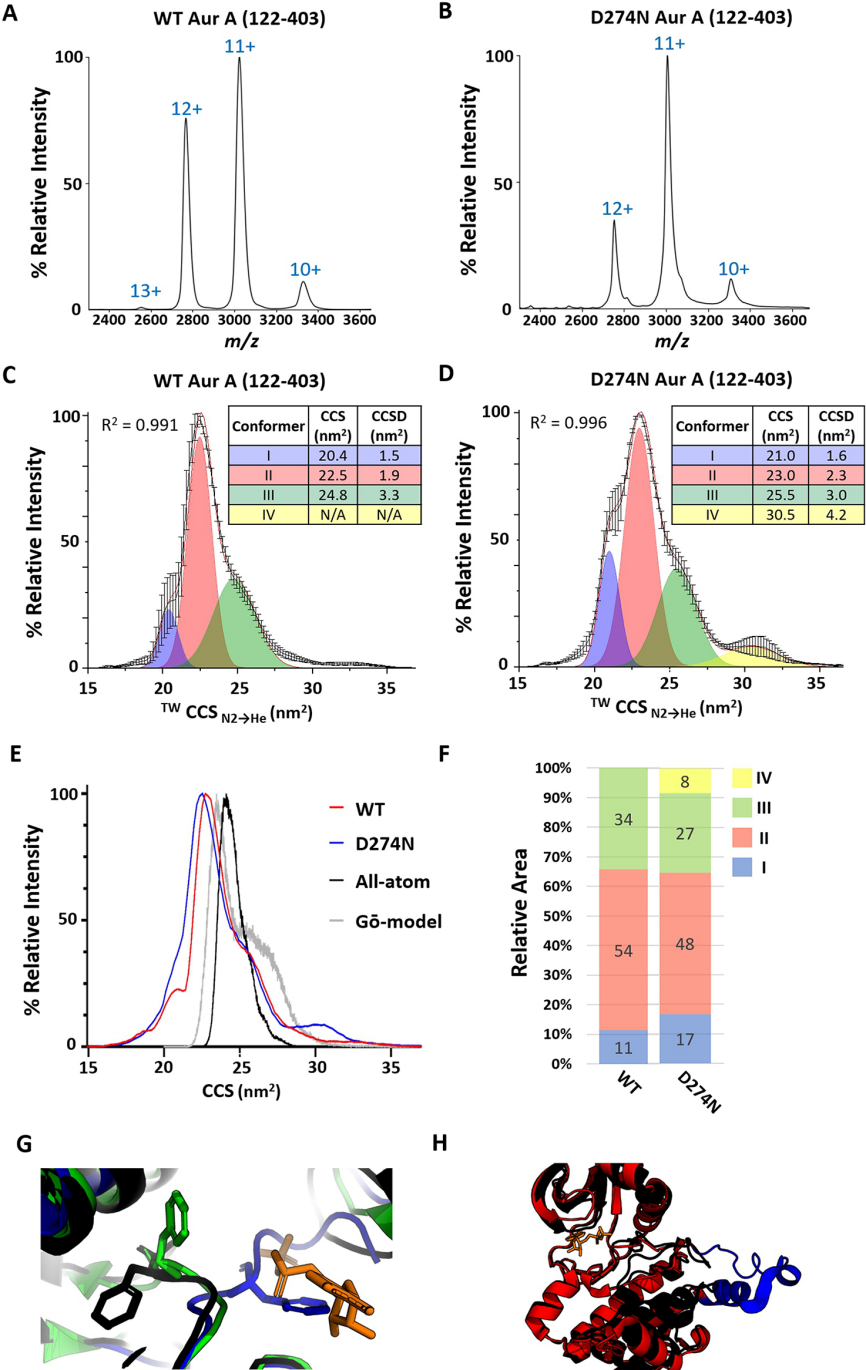
Active phosphorylated Aur A (122–403) is more conformationally compact than inactive nonphosphorylated Aur A. Native ESI mass spectrum of phosphorylated active WT (A) or nonphosphorylated inactive D274N (B) Aur A (122–403). (C–E) ^TW^CCS_N2→He_ for the [M + 11H]^11+^ species of WT (C) or D274N (D) Aur A (122–403). The red line is the average of three independent replicates. Black error bars representing the S.D. Gaussian fitting was performed using the Fit Peaks Pro function in Origin (Version 2021b), with *R*^2^ values listed. (E) Overlaid ^TW^CCS_N2→He_ for WT (red), D274N (blue) Aur A, and an overall distribution from all-atom simulations (black) and Go̅-model (gray). (F) Percentage area of the four different conformational states (as determined by Gaussian fitting in C,D): I (blue), II (red), III (green), IV (yellow) for WT and D274N Aur A (122–403). Average % area presented from three individual experiments. (G) Zoomed-in view showing the position of the Phe side-chain in select example crystal structures: DFG-in (black, 1OL7), DFG-up (green, 5L8K), DFG-out (blue, 6HJK). The ATP-binding site is marked by an ADP molecule (orange) from 1OL7; this highlights the clash with the DFG-out Phe. (H) Overlay of crystal structures from 1OL7 (black) and 4C3P (red). Each 4C3P Aur A monomer exhibits a displaced A-loop and αEF helix (colored blue) compared to other Aur A crystal structures.

The charge state distribution of both WT and D274N Aur A following native MS was relatively compact (Figure 2A,B), with 11+ and 12+ charge states of WT Aur A being observed predominantly. IM-MS analysis of the major 11+ charge state yielded a broad ^TW^CCS_N2→He_ distribution for both protein species (Figure 2C,D), with the weighted average CCS value for nonphosphorylated Aur A being marginally smaller (22.3 nm^2^) than that for the active phosphorylated Aur A (23.9 nm^2^) kinase. However, the half-height width of the CCS distribution (CCSD) of inactive D274N Aur A was much broader than that observed for the active enzyme, indicating greater conformational flexibility of the nonphosphorylated protein (Figure 2E).

Gaussian fitting of these CCS data revealed three partially overlapping conformers (termed I, II, III), with an additional fourth (IV), larger conformational state of relatively low abundance for D274N Aur A (Figure 2C,D). For ease of comparison, these data are also presented as weighted distributions for each conformer (Figure 2F). The CCS and the CCSD values of the three primary conformers (conformers I, II, and III) for both proteins were within the 3% variance generally observed with these types of native IM-MS experiments suggesting that these conformational states are likely to be analogous between the active and inactive forms of Aur A. The greater conformational flexibility of D274N compared with WT protein was explained by (i) the increase in relative abundance of conformer I for D274N AurA and (ii) the additional conformer IV that was not observed in the WT protein. These initial observations suggest that phosphorylation of Aur A serves to partially constrain the conformational landscape that this protein can adopt.

### Modeling Suggests That the Major Experimental Conformers Relate to Open/Closed States Rather than Different DFG-Motif Conformations or Activity

In support of our experimental data, we used IMPACT to estimate CCS values from several all-atom models of Aur A (122–403)-based crystal structures of Aur A found in the protein databank (PDB), including PDB codes 1OL7,^27^5L8K,^74^ and 6HJK,^75^ as examples of different configurations of the A-loop, with the DFG motif positioned as DFG-in, DFG-up, and DFG-out, respectively (Figure 2G, Table S1). One further modeled structure of note was PDB code 4C3P, where dephosphorylated Aur A was cocrystallized as a dimer in the presence of the activating TPX2 peptide and in which the A-loop and the αEF helix adopt an extended “open” configuration and the DFG motif is positioned as DFG-in.^76^ Despite the difference in DFG/A-loop position, estimates of CCS values for these static structures—barring those for 4C3P—gave similar values, averaging 22.7, 22.9, and 23.3 nm^2^ for the DFG-in, DFG-up, and DFG-out groups of structures, respectively (Table S1). These values are an excellent match for those determined experimentally for conformer II. The two 4C3P-derived models, with their “open” A-loop structure, gave higher CCS values averaging 24.9 nm^2^, in line with conformer III.

To generate a dynamic picture of protein behavior, molecular dynamics (MD) simulations were initiated from each of these structural models. Each simulation provides an ensemble of structures and yields a wider distribution of CCS values (Figure S2). When considered together, the CCS distributions resemble those observed experimentally by IM-MS for conformers II and III, albeit with the two peak positions shifted to ∼24 and ∼26 nm^2^ (Figures 2E, S2). This difference of 5–10% compared to the experimentally determined values is similar to that previously reported between experimental and IMPACT-computed CCS values for other proteins.^77^ The distributions of exemplar DFG-in (1OL7), DFG-inter (5L8K), and DFG-out (6HJK) structures are virtually indistinguishable, while the higher CCS values are almost exclusively from the 4C3P (DFG-in, A-loop open) simulations (Figures 2H, S2B; Table S1). An analysis of the DFG-motif conformation for every frame of the simulation trajectories shows that, in large part, the initial DFG-motif position is maintained within each simulation (Figure S3). Further interrogation of simulated structures (Figure S4) suggests that experimental conformers II and III do not relate to different DFG-motif conformations. Instead, conformer II represents “closed” kinase configurations, where the A-loop is inward facing, while conformer III represents the “open” configurations, one example being the dislocation of the A-loop and αEF helix as observed in 4C3P. The broader CCSD of conformer III (Figure 2C,D) suggests that this may be made up of multiple “open” configurations that cannot be resolved.

Enhanced conformation sampling at the expense of chemical detail can be achieved through use of a much-simplified, structure-based “Go̅-model”, where each residue is considered as a single “bead” and the only stabilizing interactions are those from contacts made in the initial structure.^70^ Individual Go̅-model simulations based on the1OL7crystal structure gave rise to reproducible CCS distributions that again match well with the experimental profile for conformers II and III (Figures 2D,E, S5). Further analyses of the residue contact and flexibility (RMSD), and by clustering the Go̅-model simulated structures, again suggest that conformer III could be composed of configurations with a mobile and extended A-loop but also suggest a significant contribution from configurations with a dynamic unfolding N-terminus (Figure S5).

Interestingly, none of the MD simulations reveal conformations equivalent to either conformer I or conformer IV as observed by IM-MS, suggesting that these extremes in the conformational landscape are not represented in the Protein Data Bank and may arise due to dynamic changes not permissible in crystal structures.

### TPX2 Binding Alters the Conformational Landscape of Both Phosphorylated and Nonphosphorylated Aur A

**Figure 3:**
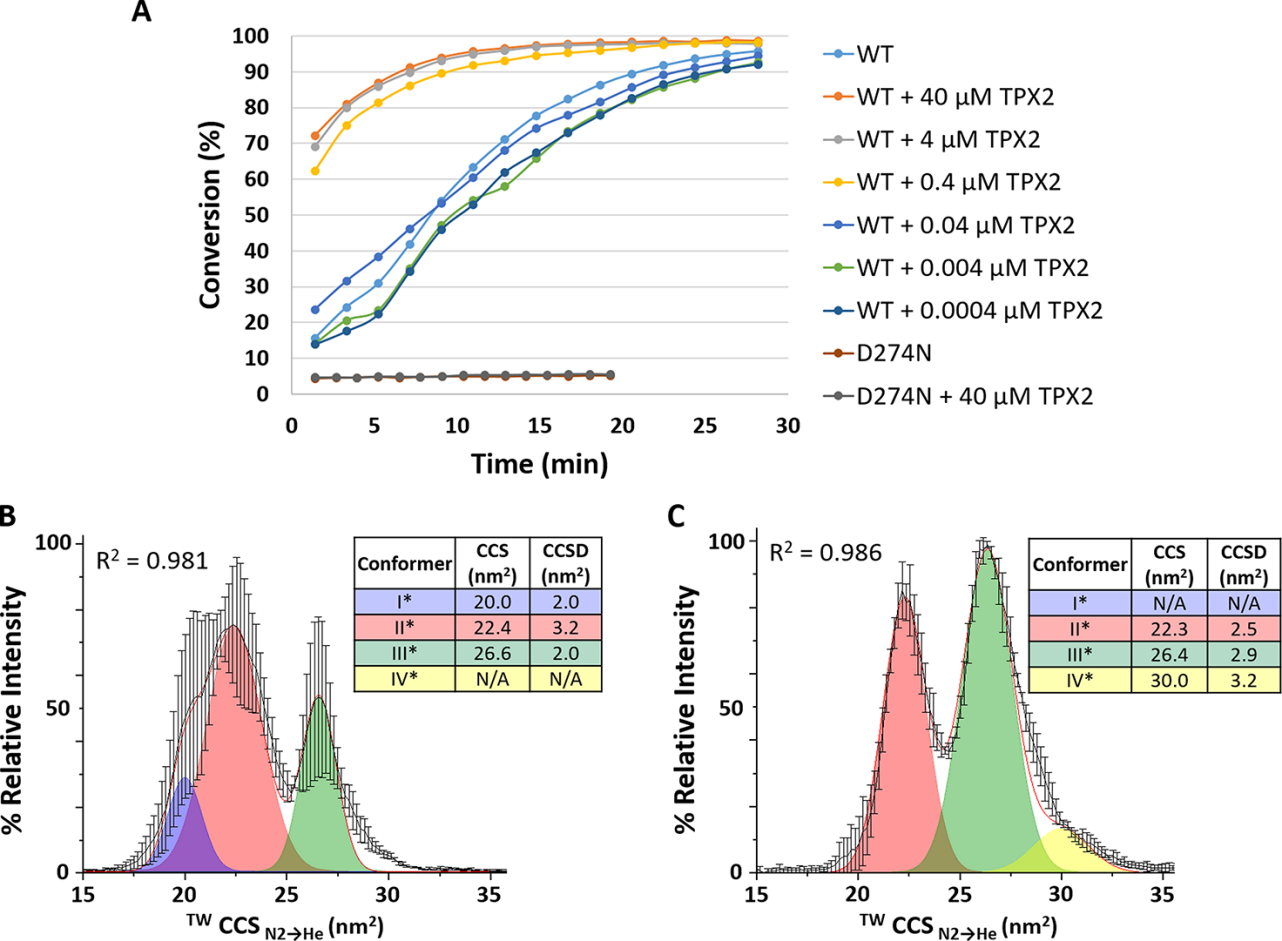
Aur-A-activating TPX2 peptide alters the conformational landscape of both phosphorylated and nonphosphorylated Aur A (122–403). (A) *In vitro* peptide-based Aur A kinase assays using 5 μM WT or D274N Aur A in the presence of the minimal activating TPX2 peptide at the indicated concentrations. (B,C) ^TW^CCS_N2→He_ of the [M + 11H]^11+^ species of WT phosphorylated active (B) or D274N nonphosphorylated inactive (C) Aur A in the presence of a 10 M excess of the minimal TPX2 peptide. The red line is the average of three independent replicates. Black error bars representing the S.D. Gaussian fitting was performed using the Fit Peaks Pro function in Origin (Version 2021b), with *R*^2^ values listed.

Binding of the minimal TPX2 peptide (1–43) to phosphorylated Aur A (122–403) has previously been shown to stabilize the active conformation of Aur A *in vitro*, interacting with the N-terminal lobe of Aur A (thereby stabilizing the position of the C-helix), and secondarily stabilize the A-loop.^78^ We thus investigated the effect of a minimal TPX2 peptide that activates Aur A on the conformational landscape of both the active and inactive forms of Aur A. Binding of the TPX2 peptide (1–43) to WT Aur A, which increased its activity ∼4-fold (Figure 3A), induced marked differences in its conformational profile (Figure 3B). Like Aur A alone, Gaussian fitting of the CCS profile of TPX2-bound Aur A revealed four conformational states, which we termed I*, II*, III*, IV*.

Although the mean weighted CCS values of the two smallest conformational states of TPX2-bound WT Aur A are comparable with the protein alone, the conformational flexibility (CCSD) of both these states increases. Further evaluation of conformer II*, and noting the broad CCSD, suggests that this may be representative of multiple configurations that are not separable under these conditions (Figure 3B). Notably, conformer III* is of higher relative abundance and exhibits a ∼7% larger CCS (and smaller CCSD) than conformer III for unbound WT Aur A, suggesting that the activating TPX2 peptide may target and stabilize this third conformational state, which we hypothesize represents the “open” Aur A configuration. Our observation of a distinct conformational topology for active Aur A in the presence of TPX2 contrasts with previous crystallographic studies that reported no global conformation change due to TPX2 (peptide) binding (with TPX2 docking into a hydrophobic groove in Aur A^27^), suggesting some solid-phase constraint of structure. Binding of the TPX2 peptide to the inactive nonphosphorylated Aur A yielded two primary CCS distributions (Figure 3C), fitting to three Gaussian peaks: II*, III*, and IV*. The CCS of conformer III* was the same for both forms of TPX2-bound Aur A, albeit at much higher abundance (and with a larger CCSD) in D274N Aur A than WT. Conformer I* was absent for D274N Aur A, while conformers IV and IV* were comparable. Interestingly, the CCS for III* is similar to that generated following the IMPACT all-atom simulation of 4C3P, the TPX2-bound “DFG-in” Aur A where the A-loop is open (Table S1; Figure S3A,B). Our data are thus consistent with TPX2-mediated Aur A activation via an “open” conformation of the A-loop that promotes autophosphorylation in trans.^76^

### Active Phosphorylated Aur A Is Less Kinetically Stable than Inactive Nonphosphorylated Protein

**Figure 4:**
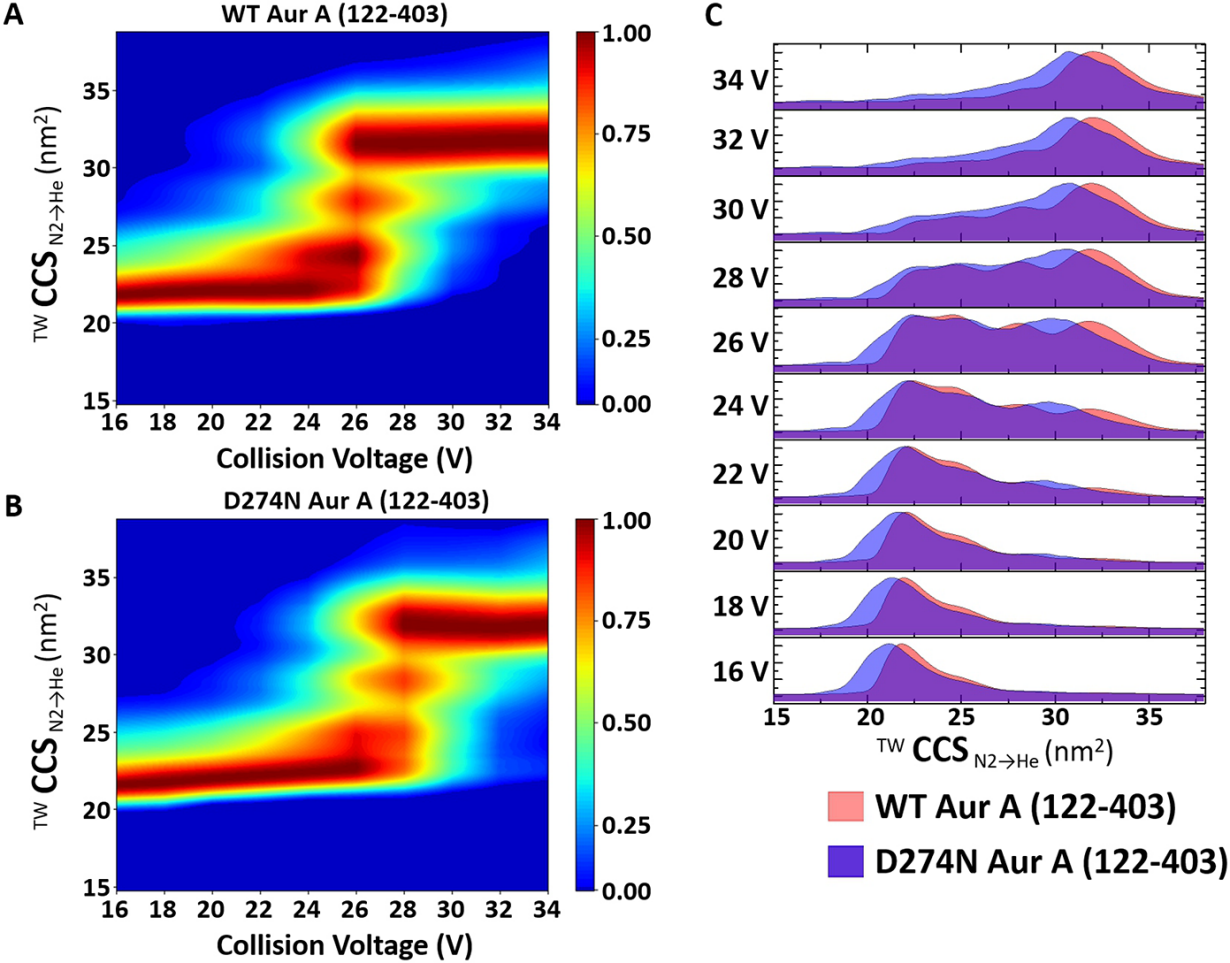
Active Aur A (122–403) is less kinetically stable than inactive Aur A. Collision-induced unfolding profiles for the isolated 11+ charge state of WT (A) and D274N (B) Aur A (122–403) (or overlaid in (C)). Stepped collision energy was applied between 16 and 34 V in 2 V intervals. Data analysis was carried out in MassLynx 4.1, (A,B) generating heat-maps using CIUSuite 2 and (C) mountain plots using Origin (Version 2016 64Bit). Presented are data from an average of three independent experiments.

To better understand relative differences in conformation stability of active phosphorylated Aur A compared with its inactive counterpart, we performed collision-induced unfolding (CIU), comparing the CCS of WT versus D274N Aur A at different collision energies (CEs) (Figure 4). The applied CE was sufficient to promote protein unfolding but not to induce protein fragmentation.

Comparing these CIU profiles provides information on relative protein kinetic stability, as the activated ions generated at each CE are trapped in a defined conformational state.^56,79−82^Figure 4A,B depicts the CIU fingerprints for WT and D274N Aur A, respectively, and a direct comparison of the conformational landscapes adopted by these two proteins at each stepped CE value is presented in Figure 4C. Four main CIU features were observed: the initial conformers (as represented in Figure 2C,D), two partially unfolded intermediates (ranging from ∼24–28 nm^2^), and final stable “unfolded” states between ∼31–33 nm^2^. Similar to the observed differences in conformational space adopted under native conditions, the final “unfolded” inactive nonphosphorylated Aur A had a larger CCSD, indicative of greater conformational flexibility. It is also interesting to note that the CE required to initiate unfolding, and to transition between the partially unfolded intermediates, was lower for active Aur A than was required for D274N (∼24 versus ∼26 V, respectively).

Overall, these data suggest that active Aur A is less conformationally dynamic than Aur A in a nonphosphorylated inactive state and that it is also less stable than the inactive protein. This gas-phase kinetic stability data agrees with the liquid-phase thermostability data generated using DSF (Figure 1A), where the *T*_m_ value (50% unfolding) was 41.7 °C for WT Aur A compared with 49.6 °C for D274N Aur A. Similar findings for solution stability have also been reported elsewhere.^83,84^

### Exposure to Small Molecule Inhibitors Alters the Conformational Distribution of Active Aur A

**Figure 5:**
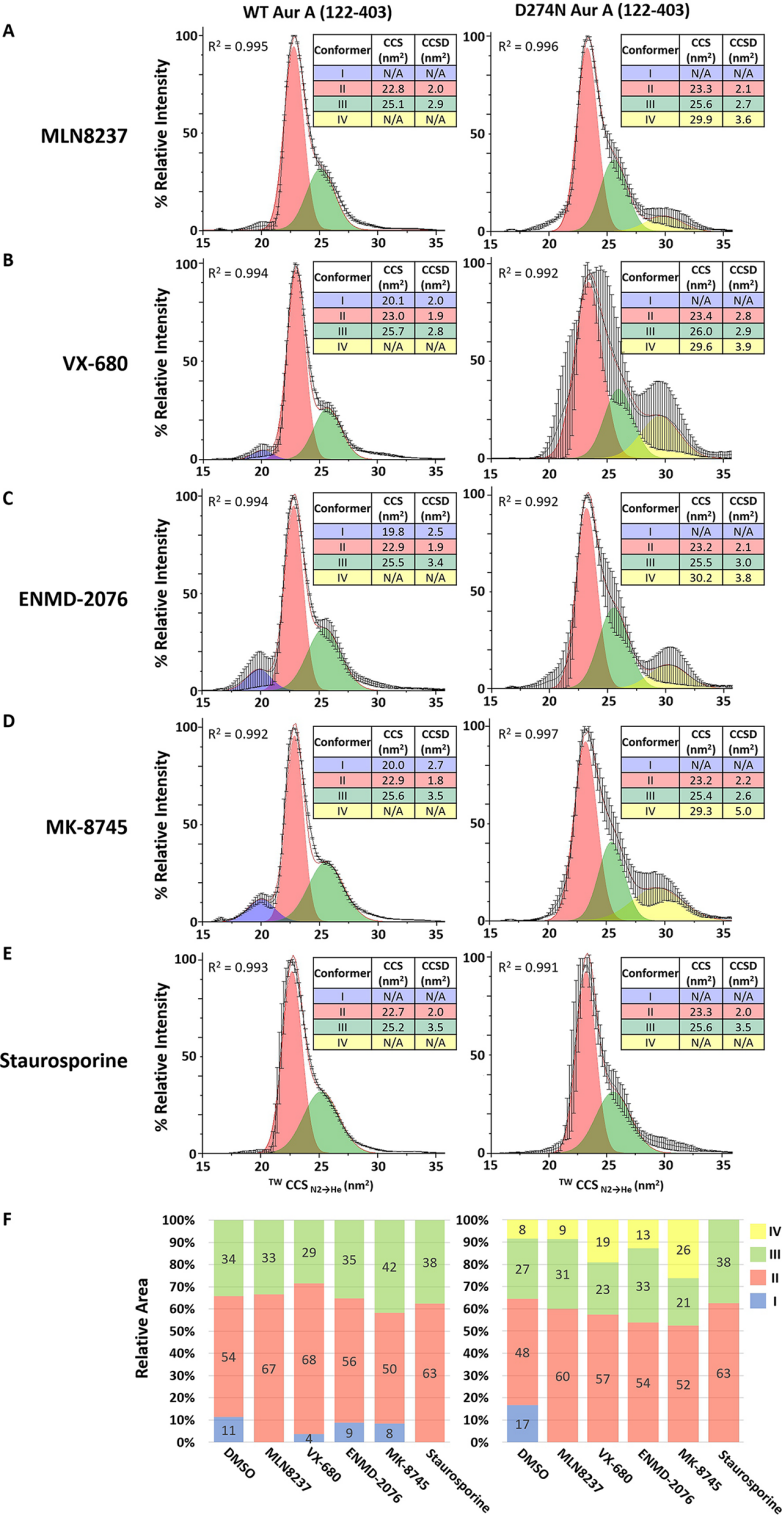
IM-MS of inhibitor-bound Aurora A (122–403). ^TW^CCS_N2→He_ of the [M + 11H]^11+^ species of WT phosphorylated active (left) or D274N nonphosphorylated inactive (right) Aur A (122–403) in the presence of a 10-fold M excess of (A) MLN8237, (B) VX-680, (C) ENMD-2076, (D) MK-8745, or (E) staurosporine. The red line is the average of three independent replicates. Black error bars representing the S.D. Gaussian fitting was performed using the Fit Peaks Pro function in Origin (Version 2021b), with *R*^2^ values listed. (F) Percentage area of the four different conformational states (as determined by Gaussian fitting): I (blue), II (red), III (green), IV (yellow) for WT (left) and D274N (right) Aur A (122–403) in DMSO control or in the presence of the different inhibitors as indicated. Average % area presented from three individual experiments.

To investigate whether we can distinguish modes of small molecule binding to Aur A by IM-MS, we next evaluated the conformational profiles of active and inactive Aur A in the presence of a panel of Aur A inhibitors (Table S2, Figure 5; Figure S7). Based on a recent analysis, ENMD-2076 should favor a DFG-in mode, whereas MK-8745 is expected to favor DFG-out. MLN8237 and VX-680 are believed to adopt a partial DFG-out position.^50^ We also investigated the structural effects induced in the presence of the generic type I protein kinase inhibitor staurosporine, which typically binds in a DFG-in conformation. The CCS/CCSD data for each of the (up to) four conformational states (Figure S6) as well as their relative proportion across the conformational landscape (as determined by Gaussian fitting of the CCS profiles for the inhibitor-bound/unbound Aur A) are also depicted as proportional plots (Figure 5F), making differences in the relative abundance of these conformational states (and their relative flexibility) easier to compare.

While distinct from unbound active Aur A, the conformational landscapes observed upon binding of the Aur A inhibitors were similar, with comparable CCS and CCSD values for the (up to) four conformers defined by Gaussian fitting of the IMS profile. Comparison with unbound active Aur A revealed a decrease in the relative abundance of conformer I in the presence of these small molecules, particularly for staurosporine and the partial DFG-out inhibitors MLN8237 and VX-680, with a concomitant increase in the relative abundance of conformer II. Where conformer I was observed, it exhibited a broader CCSD than apparent for unbound WT (DMSO control) Aur A (Figures 2, 5, S6).

Interestingly, D274N Aur A adopts a different conformational landscape in the presence of inhibitors and when compared with active Aur A bound to the same small molecule. Of note, the relative abundance of conformer II increases in the presence of all small molecules evaluated, with conformer I (which accounts for ∼17% for D274N Aur A alone) being absent (Figure 5). There was a noticeable difference in the ratio of conformers III and IV for the two partial DFG-in inhibitors MLN8237 and VX-680, with the proportion of conformer IV being greater for VX-680. With regard to staurosporine specifically, and in contrast to the other inhibitors, no significant difference was observed in the conformational landscape adopted by either WT or D274N Aur A.

### Active Aur A Is Stabilized to Varying Extents in the Presence of Different Small Molecule Inhibitors

**Figure 6:**
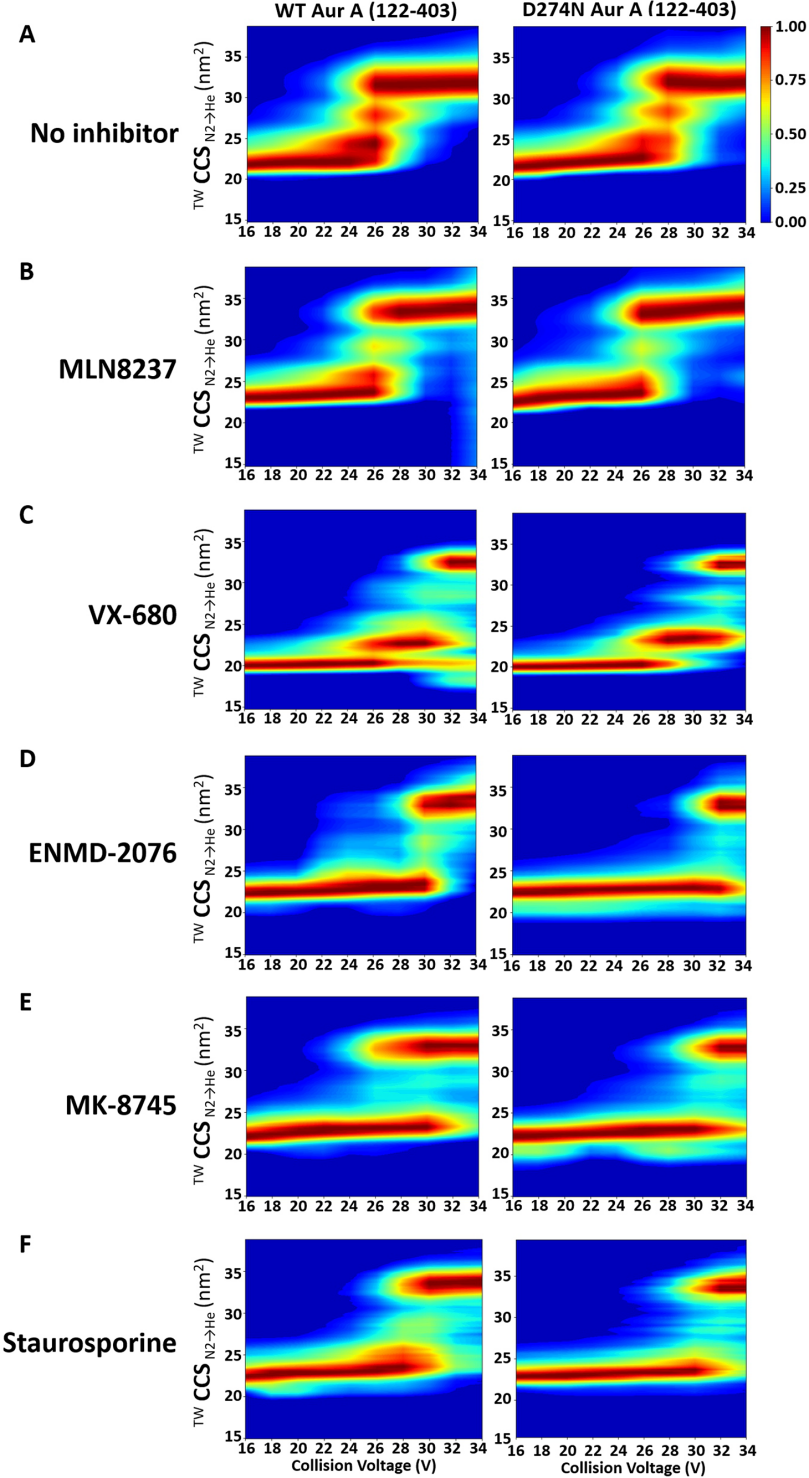
Collision-induced unfolding profiles of inhibitor-bound Aur A. The isolated 11+ charge state of (A) WT (left) and D274N (right) Aur A (122–403) in the presence of a 10 M excess of (B) MLN8237, (C) VX-680, (D) ENMD-2076, (E) MK-8745, or (F) staurosporine was subject to CIU using a stepped collision energy between 16 and 34 V (2 V intervals). Data analysis was carried out in MassLynx 4.1 (generating heat-maps using CIUSuite 2). Presented are data from a single experiment, representative of the data from independent triplicate analyses.

The lack of marked difference in the conformational landscapes of active Aur A when bound to the different types of inhibitors prompted us to explore the kinetic stability of these complexes by CIU (Figure 6; Figures S7, S8).

As can be seen from the CIU profiles, the different inhibitors had pronounced effects on the relative kinetic stability of both active and inactive Aur A. While the final stable “unfolded” structures for all the inhibitor-bound forms of WT Aur A (recorded at 34 V) approached a CCS value of ∼33–35 nm^2^, the energy required to initiate unfolding, the conformational states adopted during unfolding were markedly different (Figures 6, S7, S8). Of all the inhibitors evaluated, the unfolding profile of MLN8237-bound Aur A (active and inactive) was most similar to that of the unbound protein (Figures 6, S8). MLN8237 had little apparent effect on the kinetic stability of Aur A, as determined by the comparable CE required to induce unfolding.

Notably, the CCS of the final unfolded conformation of MLN8237-bound Aur A was larger than for the unbound form, and the relative abundance of the partially unfolded transition states was lower (Figure 6A,B; Figure S7), suggesting that the partially unfolded intermediate states were marginally less stable in the presence of MLN8237. Binding of the other partial DFG-out inhibitor, VX-680, stabilized the active enzyme, requiring a higher CE to initiate unfolding (Figure 6C). Different (more compact) transition and final states (exhibiting reduced CCSD) were also observed for VX-680-Aur A compared with unbound protein, including a particularly stable partially unfolded intermediate at ∼22.7 nm^2^. There was also some evidence of inhibitor-induced compaction during CIU of WT Aur A, with species of CCS value <20 nm^2^ being observed (Figure 6; Figure S7).

Both ENMD-2076 and MK-8745 transitioned from their native folded state to a stable “unfolded” conformer with limited observable partially unfolded intermediates, albeit with major differences in the kinetic energy required to initiate the process (Figure 6; Figure S7). ENMD-2076 induced the greatest stabilization in WT Aur A, requiring ∼30 V to unfold (Figure 6D). Although the original conformational states were retained in MK8745-bound Aur A until ∼30 V, transition to the final “unfolded” state was evident by 24 V, with the protein simultaneously adopting two distinct configurations. This difference in unfolding topology for Aur A in the presence of the DFG-out and DFG-in inhibitors was not apparent for the inactive D274N Aur A. Indeed, the CIU profiles for inactive Aur A with either ENMD-2076 or MK-8745 (or staurosporine) were essentially identical.

**Figure 7:**
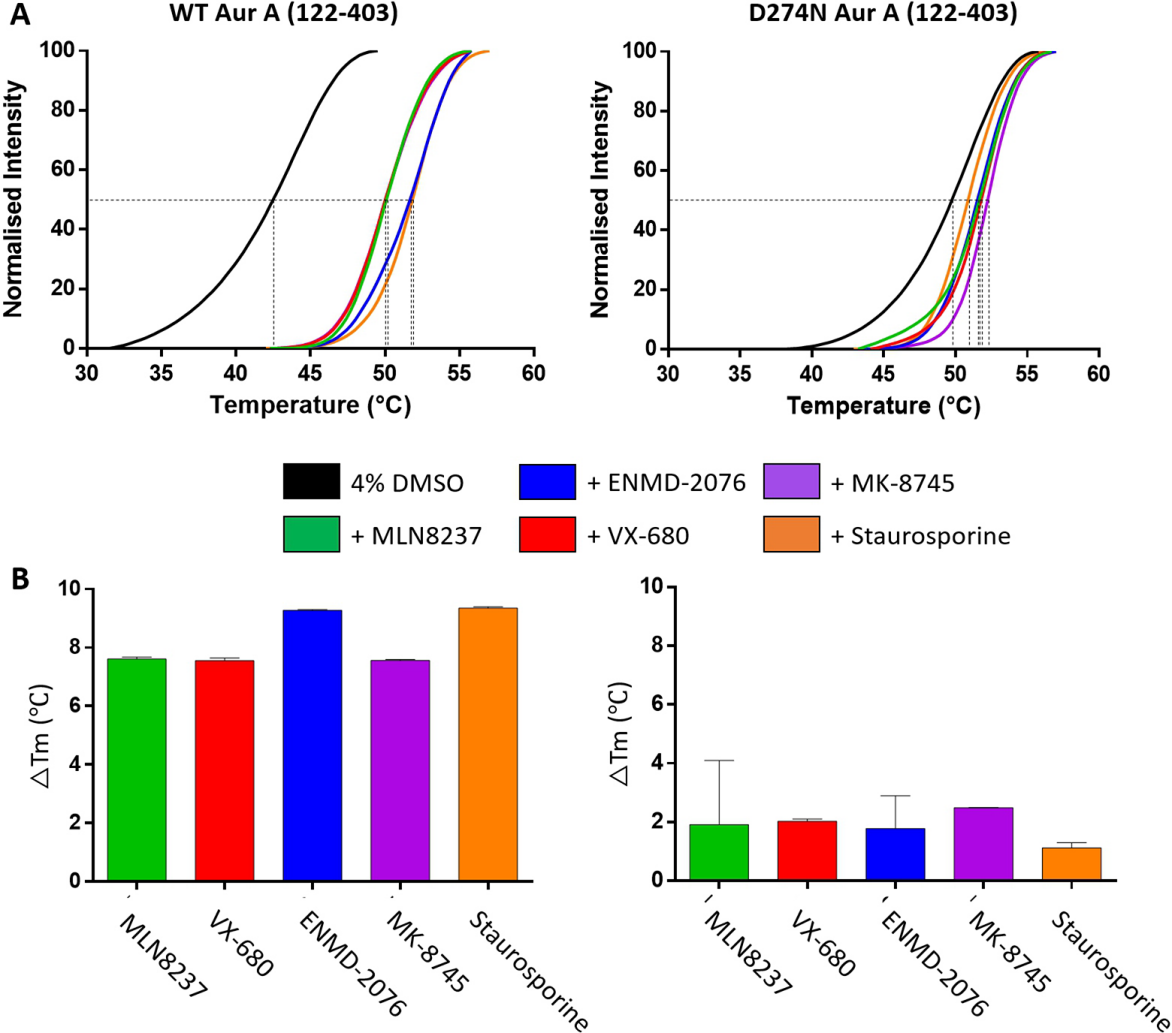
Inhibitor-induced complexation stabilizes both catalytically active and inactive Aur A. (A) DSF thermal stability assay with 5 μM Aur A + 4% DMSO (black), in the presence of 40 μM of each inhibitor. (B) Difference in melting temperature (Δ*T*_m_) relative to 4% DMSO control is presented for both WT and D274N Aur A (122–403).

Comparative thermal stability profiling of unbound versus inhibitor-bound phosphorylated Aur A by DSF (Figure 7) revealed similar unfolding profiles for WT Aur A in the presence of MLN8237, VX-680, or MK-8745, with an increase in *T*_m_ of >7.5 °C. ENMD-2076 and staurosporine induced slightly greater stabilization, with a Δ*T*_m_ of >9.2 °C. In the case of MLN8237, although we observed thermal stabilization, there was little difference in the kinetic energy required to initiate unfolding as determined by CIU. However, the CE required to reach the final stable “unfolded” configuration of WT Aur A was higher in the presence of MLN8237, suggesting that *T*_m_ measurements are likely more representative of the energy required to reach a stable unfolded state. This hypothesis holds true for all inhibitor-bound forms of WT Aur A, with the exception of VX-680, but not for inhibitors bound to inactive Aur A. While Δ*T*_m_ values associated with inhibitor-bound D274N Aur A were relatively small (∼<2.5 °C) (Figure 7), protein unfolding required a higher CE for all bound forms. Indeed, with the exception of MK-8745, there was little difference in unfolding profiles and the CEs required to induce unfolding for a given inhibitor, between active and inactive Aur A (Figures 6, S7), as exemplified by the comparison of the extracted partially unfolded profiles obtained at a CE of 26 V (Figure S8).^83,85,86^

## Discussion and Conclusions

In this study, we exploited IM-MS to explore changes and differences in the conformational landscape of purified Aur A (122–403) in active (phosphorylated) and inactive (nonphosphorylated) forms. For the first time, we also examined the effect of an activating TPX2 peptide on Aur A structural dynamics and evaluated the effects of different classes of small molecule Aur A inhibitors. Gaussian fitting of our IM-MS data revealed up to four conformational states for Aur A, subtle variations in which (primarily their relative ratio) were dependent on Aur A activation status and inhibitor binding. Active phosphorylated Aur A exhibited reduced conformational dynamics and stability than the catalytically inactive protein (Figures 1 and 2), as determined by both DSF thermal stability and CIU IM-MS experiments. Additionally, our experimentally derived CCS values for active Aur A were supported by molecular modeling approaches.

Based on CCS distributions generated from molecular simulations of Aur A in different conformations, we propose that conformer II (at ∼23 nm^2^) is representative of a “closed” state (be it DFG-in/up/out), where the A-loop is inward facing, while conformer III represents one or more “open” kinase configurations where the A-loop extends out.

At the outset of this study, we hypothesized that the activation status of Aur A and the binding of different classes of small molecule inhibitor would alter the conformational landscape adopted by this protein, as has been established previously,^85,86^ for other protein kinases such as PKA,^56,87^ c-Abl,^88^ and FGFR1^89^ as well as intrinsically disordered proteins such as p53^12,90^ and Aβ40.^91^ While we do observe some differences, the effects are subtler than might be anticipated, being broadly consistent with the findings of others.^85,86^ Inhibitor-specific structural effects that have previously been shown to alter the position of the DFG loop using X-ray crystallography were hard to detect in the gas phase using IMS. This may be due either to the inherent protein flexibility in these types of experiments or a need for greater IMS resolution.

CIU analysis started to unravel specific inhibitor-induced differences in Aur A, although all inhibitors stabilized Aur A with respect to unfolding (as confirmed in solution by DSF). This effect was most marked with the DFG-in inhibitor ENMD-2076 and the partial DFG-up/inter inhibitor VX-680 (Figures 6, S7), as can be seen most clearly when we consider the difference in conformational profiles at midunfolding in the snapshot taken at CE of 26 V (Figure S8).

Overall, our CIU experiments indicate that all inhibitors evaluated resulted in kinetic stabilization of Aur A, given that higher collision energy was required to initiate unfolding, with this effect being least apparent with MLN8237 and highest with ENMD-2076 and VX-680 (Figures 5, S6). More interestingly, by application of CIU, we were able to observe differences in the relative kinetic stability of Aur A when bound to the partial DFG-out inhibitors, as opposed to either a DFG-in or a DFG-out inhibitor. Notably, the partially unfolded transition states observed for active Aur A alone or in the presence of either MLN8237 or VX-680 were absent with the other small molecules (Figure 6) suggesting that these partial DFG-out inhibitors function to “lock” Aur A into specific configurations. The effects of binding of these two inhibitors to Aur A are thus likely not only a function of the position of the DFG/P-loop but also reliant on the precise nature of the noncovalent interactions mediated by different chemical classes. We cautiously interpret this finding in the context of the DFG-up conformation, which has been observed with MLN8054 and VX-680 in complex with Aur A (PDB codes2WTVand4JBQ, respectively). Finally, we anticipate that future studies employing IM-MS and CIU may prove useful in characterizing the conformational space adopted by other druggable enzymes, including the >500 members of the human kinome.

## Supporting Information Available

Biochemical characterization of WT and D274N Aur A, additional molecular modeling and ion mobility data including CIU profiles, and details of the compounds used in this study (PDF)
