# Supplementary material for: Exploring the Conformational Landscape and Stability of Aurora A Using Ion-Mobility Mass Spectrometry and Molecular Modeling
Source: J Am Soc Mass Spectrom. 2022 Jan 31;33(3):420–35. doi: 10.1021/jasms.1c00271 (PMC9007459; doi:10.1021/jasms.1c00271)
Supplement: Supplementary file 1 — js1c00271_si_001.pdf [file js1c00271_si_001.pdf]

# Supporting Information

## Exploring the conformational landscape and stability of Aurora A using ion-mobility mass spectrometry and molecular modelling

Lauren J. Tomlinson[1, 3], Matthew Batchelor[2], Joscelyn Sarsby[1], Dominic P. Byrne[3], Philip Brownridge[1], Richard Bayliss[2], Patrick A. Eyers[3] and Claire E. Eyers[1, 3]*.

[1] Centre for Proteome Research, Department of Biochemistry & Systems Biology, Institute of Systems, Molecular & Integrative Biology, University of Liverpool, Crown Street, Liverpool, L69 7ZB, U.K.

[2] Astbury Centre for Structural Molecular Biology, School of Molecular and Cellular Biology, Faculty of Biological Sciences, University of Leeds, Leeds, LS2 9JT, U.K.

[3] Department of Biochemistry & Systems Biology, Institute of Systems, Molecular & Integrative Biology, University of Liverpool, Crown Street, Liverpool, L69 7ZB, U.K.

*Correspondence: Claire E. Eyers (ceyers@liverpool.ac.uk)

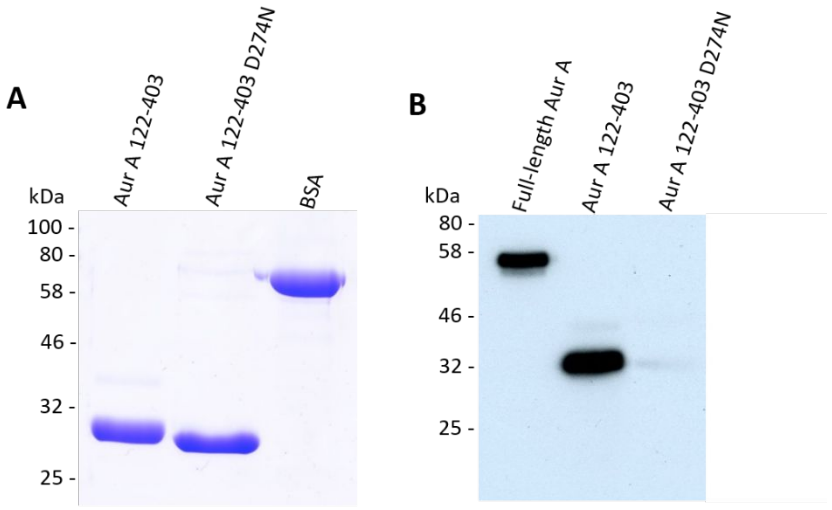


C

| Peptide sequence | Modification | Charge | m/z (Da) | DeltaM (ppm) | Ion score | ptmRS |
|---|---|---|---|---|---|---|
| EVEIQsHLR | S186 (Phospho) | 2 | 595.78 | 1.16 | 22 | 100 |
| ELQKLsKFDEQR | S226 (Phospho) | 3 | 534.26 | 1.89 | 32 | 100 |
| DIKPENLLLGsAGELK | S266 (Phospho) | 2 | 888.96 | 0.99 | 61 | 100 |
| IADFGWsVHAPSSR | S278 (Phospho) | 2 | 805.36 | 1.05 | 73 | 100 |
| RttLAGTLDYLPPEmIEGR | T287 (Phospho); T288 (Phospho); M300 (Oxidation) | 2 | 1155.01 | -3.84 | 43 | 99.75; 100 |

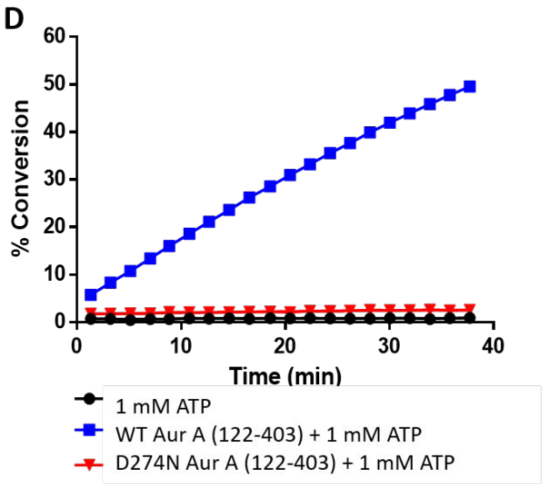


**Figure S1. Expression and purification of wild-type (WT) phosphorylated Aur A (122-403) and catalytically inactive D274N Aur A (amino acids 122-403).** (A) SDS-PAGE gel of purified WT and D274N Aur A (122-403) proteins. His-Aur A proteins were purified using a Nickel HisTrap HP column, prior to tag cleavage and gel filtration. Aur A (122-403) has a predicted MW of 32 kDa. BSA was used as a control (B) Immunoblot of full-length Aur A (1-403), WT and D274N Aur A (122-403). Phosphorylation at the activation site in the T-loop (T288) was evaluated using an anti-phospho T288 antibody (CST); 200 ng of protein was loaded per well. (C) LC-MS/MS phosphosite mapping of WT Aur A detailing the peptide sequence identified, the site(s) of modification and the confidence associated with both (MASCOT Ion score and ptmRS scores respectively). Data is representative of three independent experiments. (D) Enzyme activity assay of WT and D274N Aur A (122-403) in the presence of 1 mM ATP (10 ng protein, 2 μM substrate peptide).

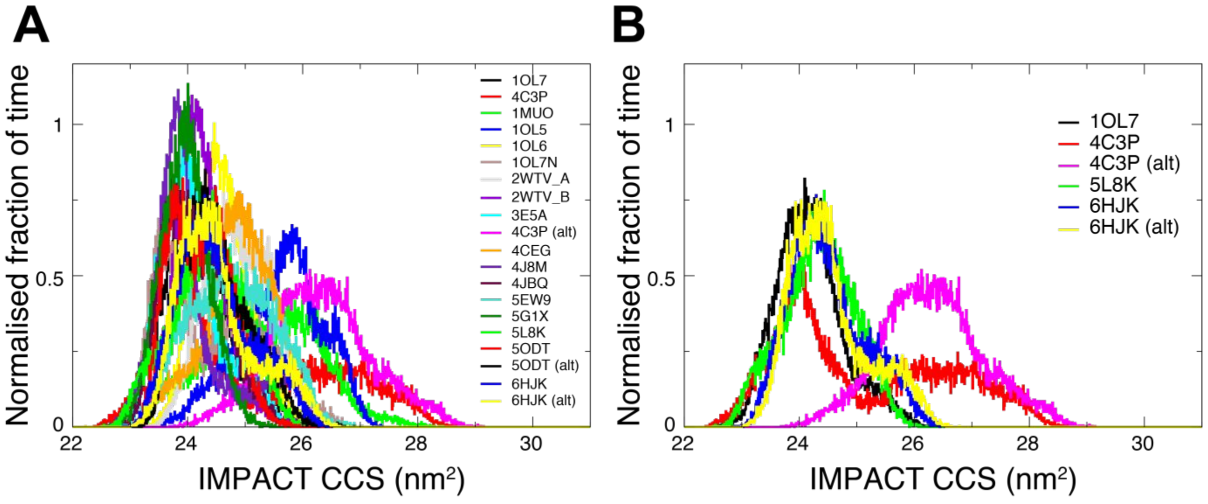


**Figure S2. Collision cross section distributions from all-atom simulations of Aur A.** Overlaid normalised distributions for (A) all simulations, and (B) example structures 1OL7 (DFG-in), 5L8K (DFG-up), 6FHK (DFG-out) and 4C3P (DFG-in, A-loop out). The bulk of trajectories contribute to a first major peak at ~24 $nm^2$ and extend between 22.5 and 27 $nm^2$. A handful of models, particularly those of 4C3P, extend the range to higher CCS values contributing to a second peak and the tail of the CCS distribution.

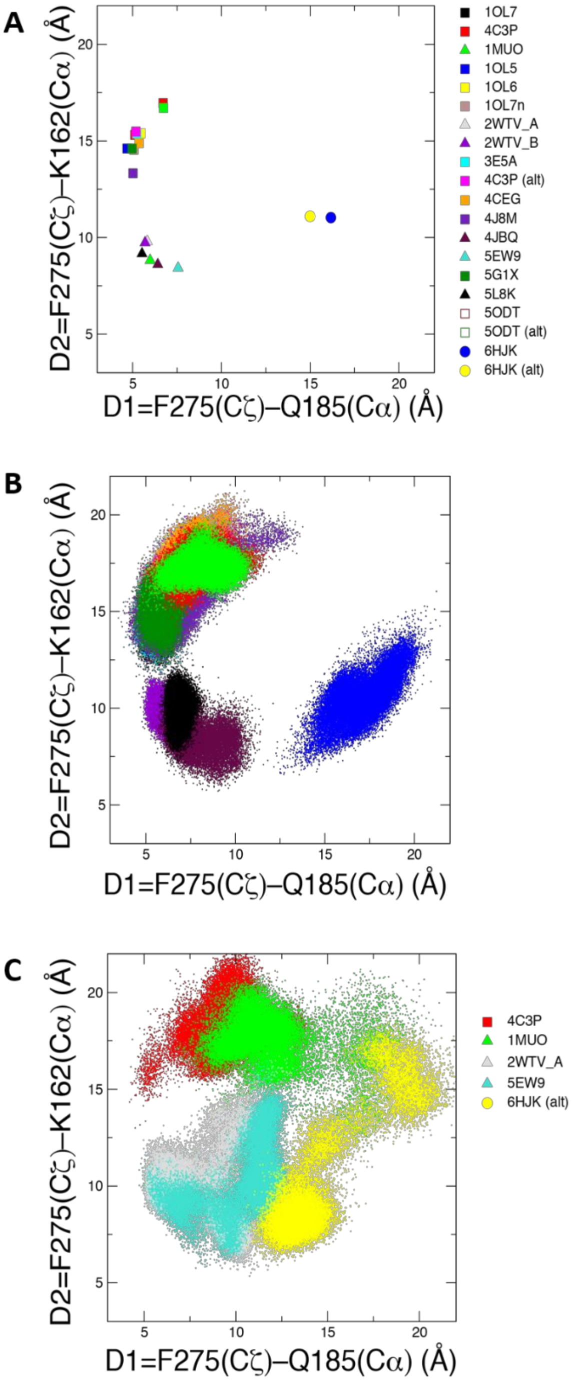


**Figure S3. Designation of DFG motif conformations using the D1/D2 criteria introduced by Modi and Dunbrack (PNAS, 2019, 10.1073/pnas.1814279116).** (A) Positions in the D1/D2 plot for each of the starting structures. Three clear groups are observed: the DFG-in group of 12 structures (squares) positioned in the upper left quadrant; the DFG-up/inter group of 6 structures (triangles) in the lower left quadrant; and the two DFG-out 6HJK structures (circles) in the lower right quadrant. (B, C) The range of the D1/D2 plot explored for each simulation. In (B), the majority of simulations remain reasonably close to their initial position and maintain their DFG conformation. In (C), five trajectories are more mobile on the D1/D2 plot and exhibit DFG motif conformations further away from their initial structure, including some conformations in the upper right quadrant of the D1/D2 plot that are not observed in kinase crystal structures.

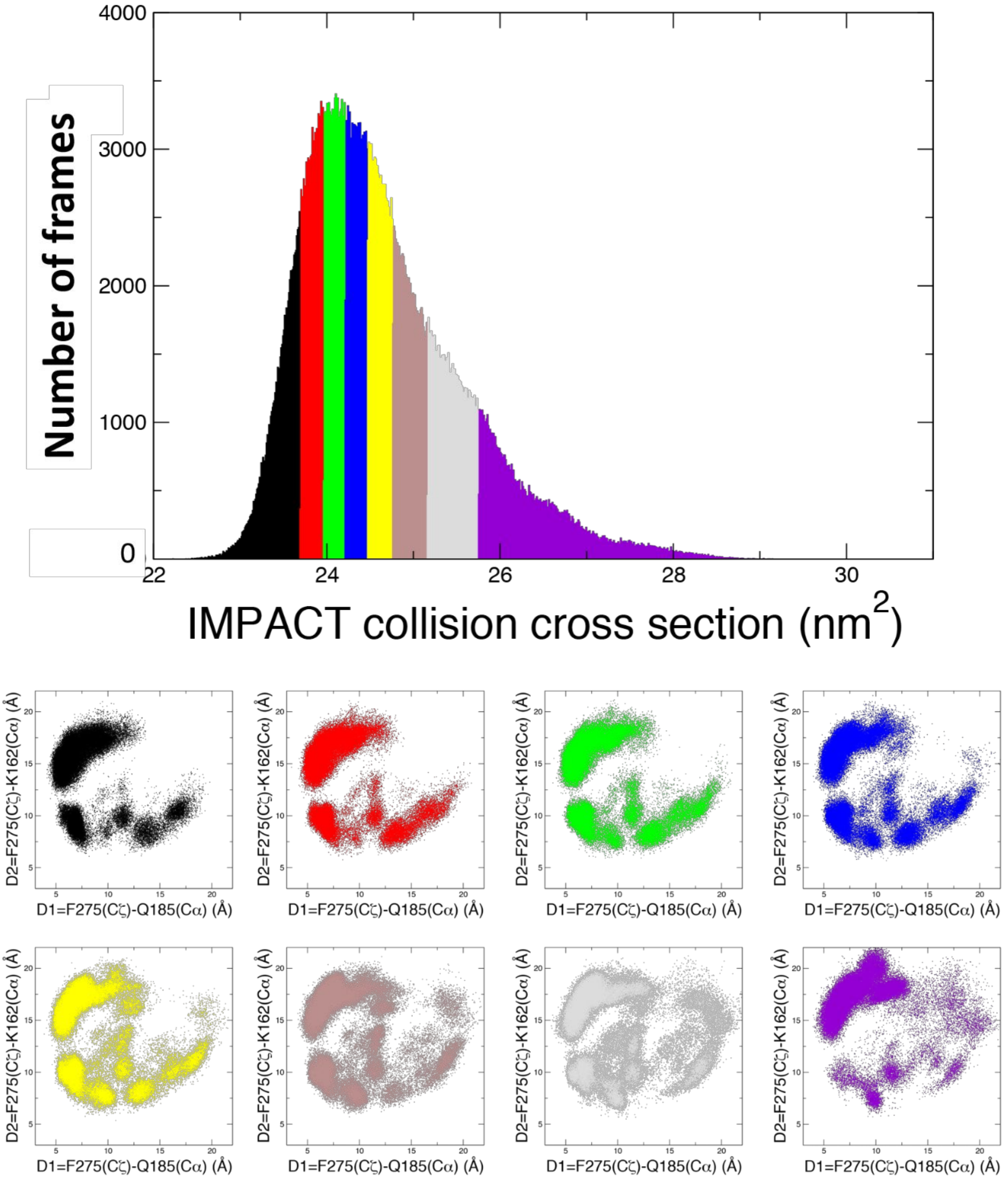


**Figure S4. Collision cross section distributions for the combined all-atom simulation trajectories of Aur A.** The distribution has been divided up into eight CCS regions with ~80,000 structures in each region. The contribution of different DFG motif conformations in each region is described using a D1/D2 plot. A high density of DFG-in models is observed in all eight regions on the CCS profile, as shown by the density of structures in the upper left quadrant of the D1/D2 diagram. DFG-in is the most common DFG motif conformer of the initial structures (12 of 20) and includes the A-loop extended 4C3P model. DFG-up/inter and DFG-out conformers are also seen across all eight regions but are less frequently observed at the highest CCS regions. The upper-right quadrant of the D1/D2 plot is more frequently occupied in the highest CCS regions, suggesting that uncategorised DFG motif conformers (not observed in crystal structures) with a more open structure contribute to the high CCS range of the profile.

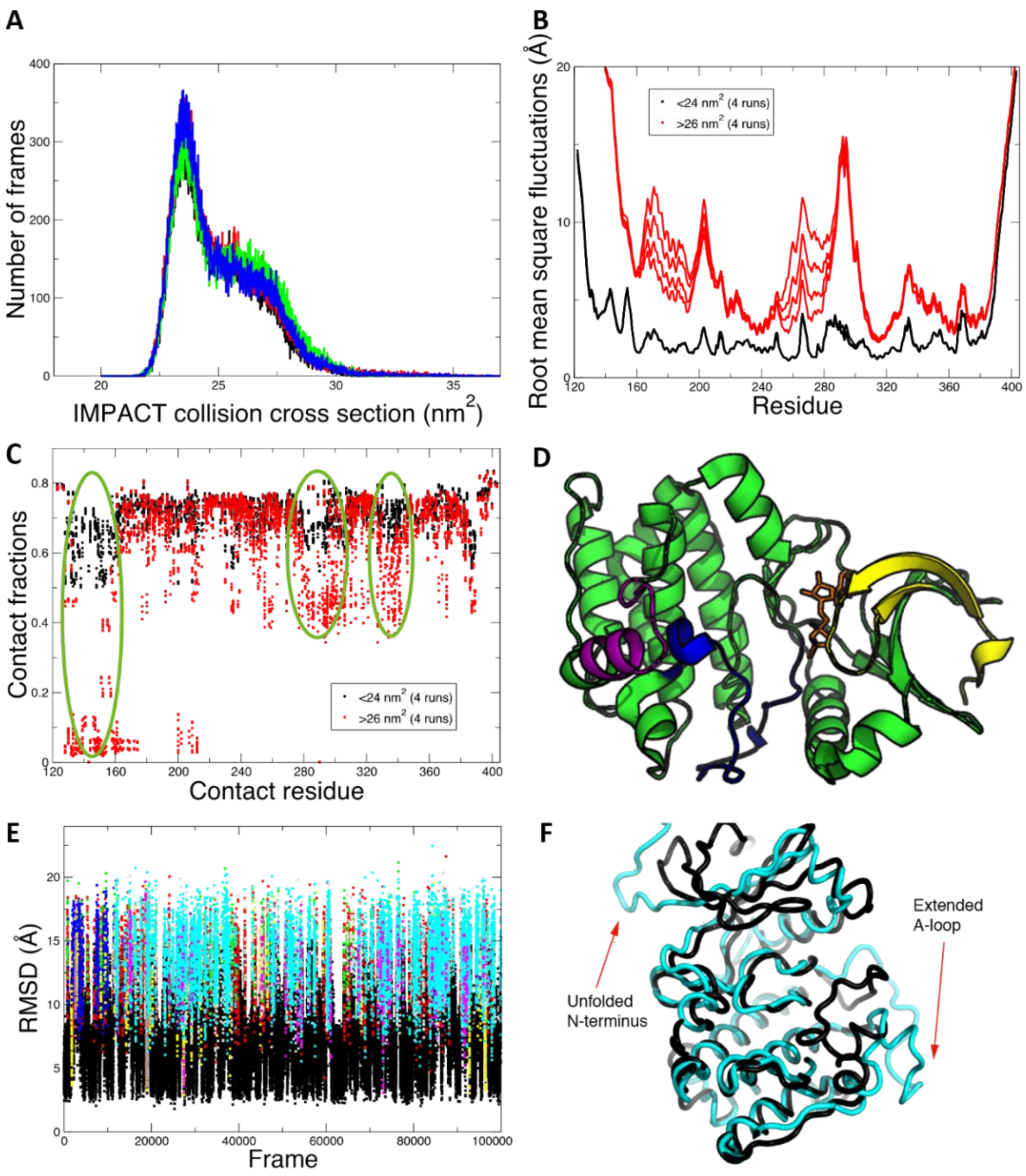


**Figure S5. Gō model simulations of Aur A produce CCS profiles similar to those of experimental IM-MS conformers II and III, and suggest that structures with an extended A-loop and unfolded N-terminus may contribute to the higher CCS (conformer III) region.** (A) Overlaid CCS distributions for 4 independent Gō model simulations display the two-component profile akin to those of the combined all-atom simulations and the experimental CCS distributions for conformer II and III. Simulation trajectory frames were separated out into first peak (<24 nm$^2$, mainly 'conformer II') and second peak (>26 nm$^2$, mainly 'conformer III') groups and then analysed to give: the root mean square fluctuations (RMSF) (B), and the native contact fractions (C). RMSF values are generally higher, marking more variable regions/structures, for the conformer III type (red) ensemble of structures compared to the conformer II-type (black) structures, but especially so in the N-lobe and around the A-loop region. Native contacts are also most likely broken for conformer III-type structures at the N-terminus, and the A-loop and contacting regions, highlighted in (D). An RMSD-based cluster analysis for one of the trajectories, gave representative structures of well-populated clusters with an unfolded N-terminus and extended A-loop region (cyan, E, F).

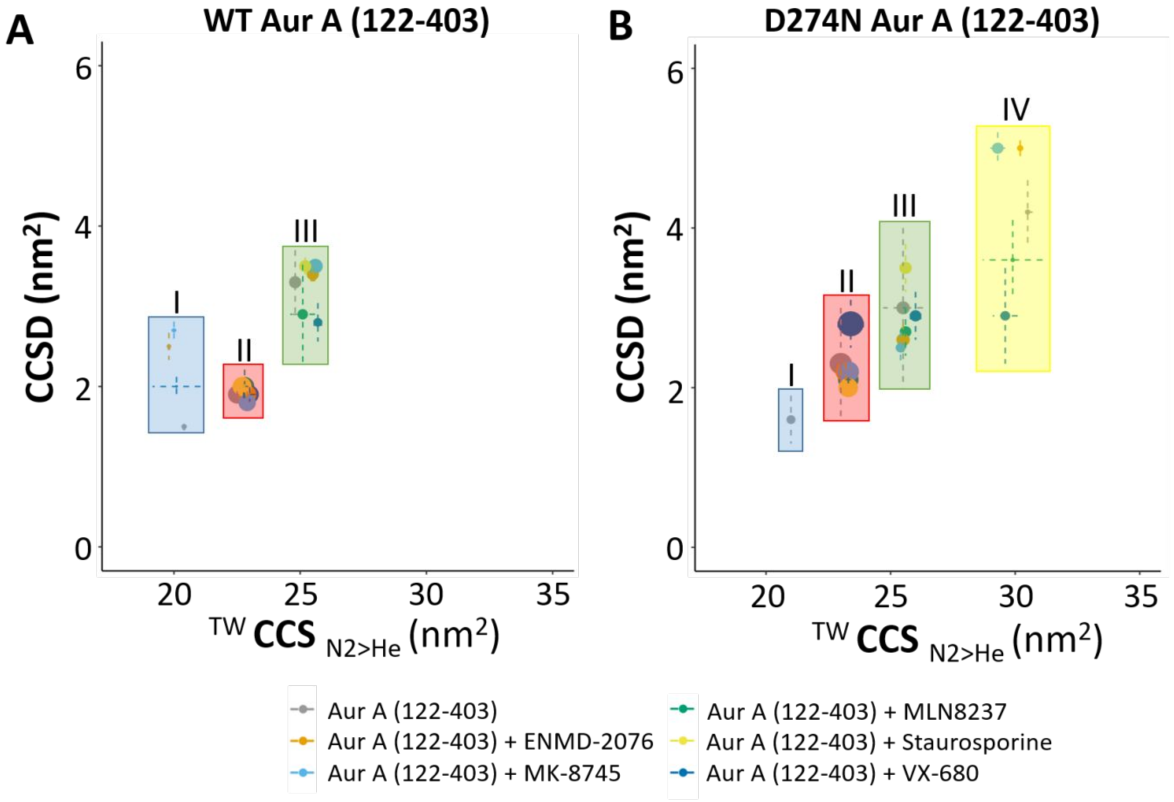


**Figure S6. Conformational space adopted by Aur A.** (A, B) Proportional scatter plots (CCS ($nm^2$) versus CCSD ($nm^2$)) for the different conformational states (as determined by Gaussian fitting in Fig. 5) for WT (A) and D274N (B) Aur A (122-403). Size of dot representative of area.

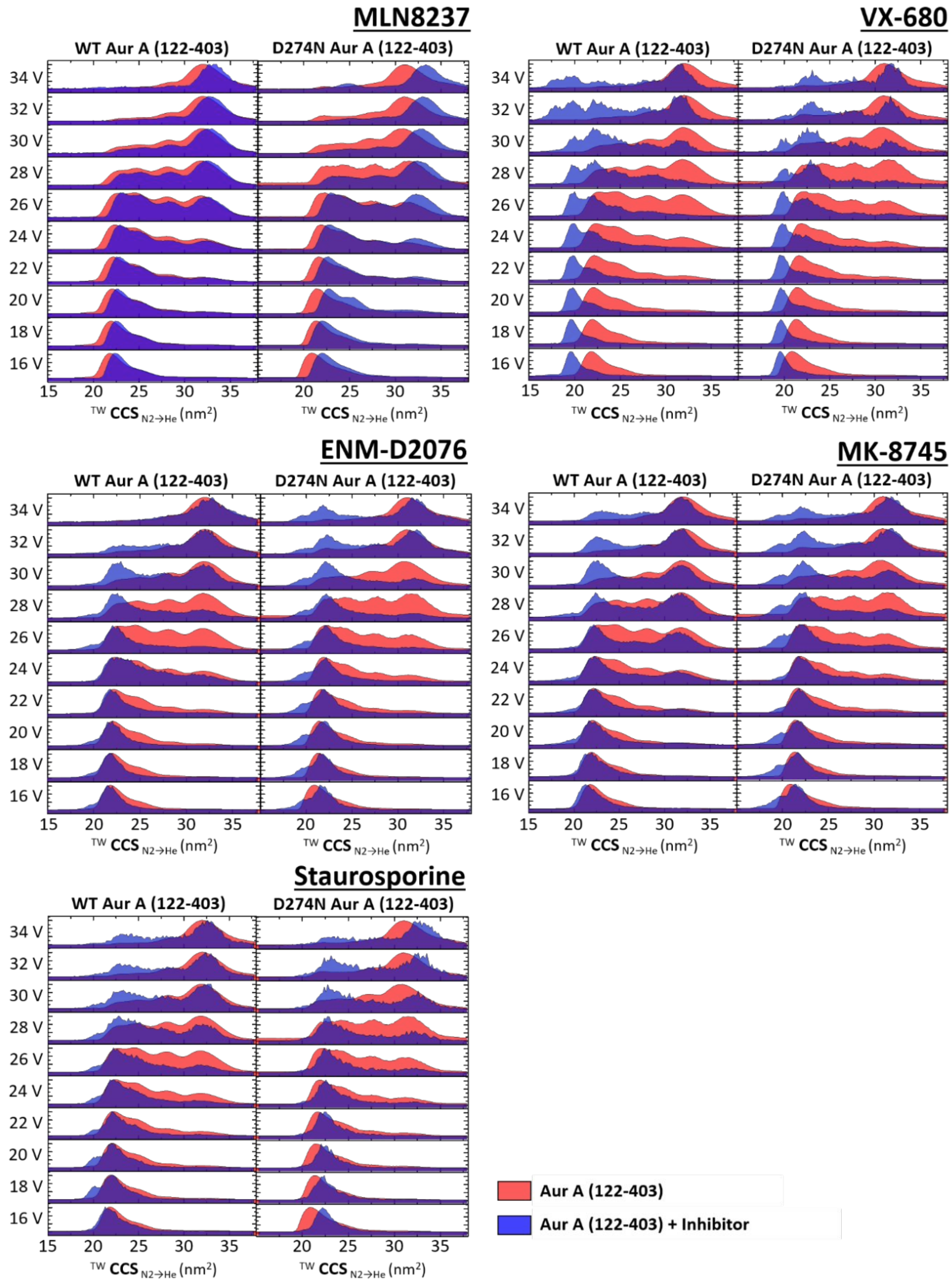


**Figure S7. Collision-induced unfolding profiles of Aur A in the absence and presence of inhibitors.** The isolated 11+ charge state of WT (left) and D274N (right) Aur A (122-403) in the presence (blue) or absence (red) of 10-fold molar excess of inhibitor were subject to CIU using a stepped collision energy (CE) between 16 and 34 V (two-volt intervals). Data analysis was carried out in MassLynx 4.1, generating mountain plots using Origin (Version 2016). Presented are data from an average of three replicates.

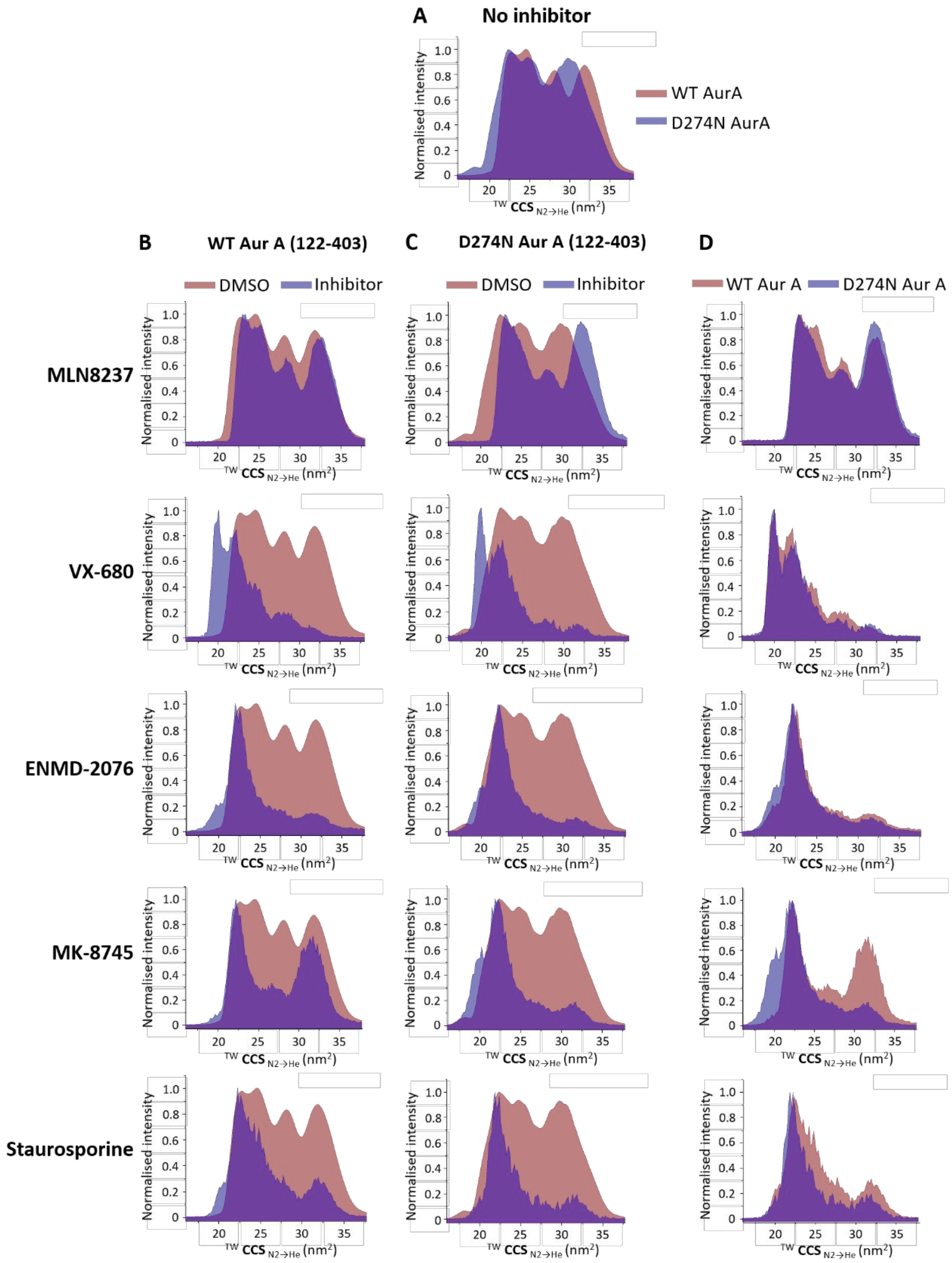


**Figure S8. Collision-induced unfolding profiles of Aur A in the absence and presence of inhibitors.** The isolated 11+ charge state at 26 V collision energy of (A) Aur A (122-403) with no inhibitor. (B) WT Aur A (122-403) and (C) D274N Aur A (122-403) in the presence (blue) or absence (red) of 10-molar excess of inhibitor. (D) Aur A (122-403) in the presence of inhibitor for WT (red) and D274N (blue). Data analysis was carried out in MassLynx 4.1 and line plots were generated using Origin (Version 2016). Presented are data from an average of three replicates.

| PDB | [1]CCS ($nm^2$) | Crystal description |
|---|---|---|
| 1OL5 | 22.6–23.0 | phos, TPX2, DFG-in |
| 1OL6 | 22.3–22.8 | dephos, D274N, DFG-in |
| 1OL7 | 22.2–22.7 | phos, DFG-in |
| 1OL7N | 22.1–22.6 | based on 1OL7, dephos, D274N, DFG-in |
| 3E5A | 22.5–22.9 | phos, VX-680, TPX2, DFG-in |
| 4C3P | 25.0–25.5 | dephos, TPX2, dimer, DFG-in, A-loop open |
| 4C3P (alt.) | 24.3–24.8 | dephos, TPX2, dimer, DFG-in, A-loop open |
| 4J8M | 23.1–23.7 | dephos, T287D, CD532, DFG-in |
| 4CEG | 21.1–21.6 | phos, C290A, C393A, DFG-in |
| 5G1X | 21.8–22.2 | phos, N-MYC, DFG-in |
| 5ODT | 23.2–23.7 | dephos, D274N, TACC3, DFG-in |
| 5ODT (alt.) | 23.9–24.3 | dephos, D274N, TACC3, DFG-in |
| 1MUO | 23.2–23.6 | dephos, adenosine, DFG-up |
| 2WTV | 23.4–23.7 | phos, MLN8054, chain A, DFG-up |
| 2WTV | 22.6–23.0 | phos, MLN8054, chain B, DFG-up |
| 4JBQ | 21.9–22.5 | dephos, VX-680, DFG-up |
| 5EW9 | 22.8–23.4 | phos, MK-5108, DFG-up |
| 5L8K | 22.3–22.7 | phos, vNAR, DFG-up |
| 6HJK | 22.9–23.4 | dephos, L210C, ASDO2, DFG-out |
| 6HJK (alt.) | 23.0–23.4 | dephos, L210C, ASDO2, DFG-out |
| | | |
| 4F5S | 39.3–40.1 | BSA, calibrant |

**Table S1. Collision cross section estimates made using IMPACT on a single initial structural model of Aur A (122–403) for each of the listed PDB codes.** DFG-in models are grouped in blue, DFG-up (or inter) models are grouped in yellow, and two alternatively built starting structures for the only available DFG-out Aur A structure (6HJK) are green.

[1] As IMPACT is a stochastic method, CCS estimates were performed 200 times on each structure to give a range of values.

| Inhibitor | Mass (Da) | Structure | Binding configuration |
|---|---|---|---|
| MLN8237 (Alisertib) | 518.92 | 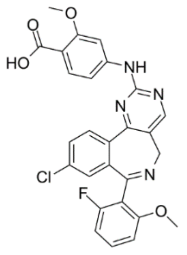 | DFG-out partial (TR-FRET)<br>Closely-related MLN8054<br>DFG-up (X-ray; PDB 2WTV)<br>DFG-in (X-ray; PDB 2WTW) |
| VX-680<br>(Tozasertib) | 464.59 | 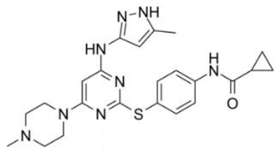 | DFG-out partial (TR-FRET)<br>DFG-in with TPX2 (X-ray; PDB 3E5A)<br>DFG-up without TPX2 (X-ray; PDB 4JBQ) |
| ENMD-2076 | 375.47 | 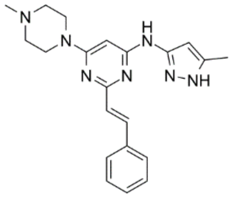 | DFG-in (TR-FRET) |
| MK-8745 | 431.91 | 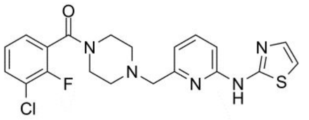 | DFG-out (TR-FRET) |
| Staurosporine | 466.53 | 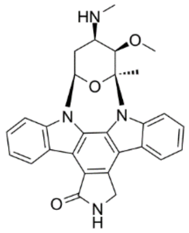 | ND |

**Table S2. Aur A inhibitors employed in this study. ND: Not Determined**
